# Perceived facilitators and barriers influencing lifestyle modification among adults with cardiovascular disease attending a cardiac clinic at a referral hospital in northern Tanzania: An exploratory qualitative study

**DOI:** 10.1371/journal.pgph.0007537

**Published:** 2026-10-27

**Authors:** Gladness N. Mkawe, Benedicto Mgala, Neema Mawi, Salome E. Buluba, Menti Ndile, Khadija Malima, Edith Tarimo, Fatina B. Ramadhani

**Affiliations:** 1 Department of Clinical Nursing, Muhimbili University of Health and Allied Sciences, Dar es Salaam, Tanzania; 2 Department of Clinical Nursing, Kilimanjaro Christian Medical Centre, Kilimanjaro, Tanzania; 3 Department of Nursing Education and Management, Muhimbili University of Health and Allied Sciences, Dar es Salaam, Tanzania; Universiti Sains Malaysia, MALAYSIA

## Abstract

Cardiovascular diseases (CVDs) are a significant cause of morbidity and mortality worldwide, with lifestyle modification being a cornerstone in their management. However, qualitative evidence on the influences that facilitate or hinder lifestyle modification practices among patients attending cardiac clinics in Tanzania remains limited. Understanding these influences is important for designing feasible, context-appropriate cardiovascular risk-reduction interventions. This study aimed to explore perceived facilitators and barriers influencing lifestyle modification among adults with cardiovascular disease attending the cardiac clinic at Kilimanjaro Christian Medical Centre (KCMC). An exploratory qualitative study was conducted between May and July 2024 among adults with CVD attending the KCMC cardiac clinic. Ten (10) participants were purposively recruited to capture variation in age, sex, and cardiovascular diagnosis. The final sample was guided by thematic saturation, reached after eight interviews and confirmed through two additional interviews. Data were collected through in-depth semi-structured interviews and analyzed manually using Braun and Clarke’s six-phase thematic analysis. The analysis generated two themes supported by sub-themes: (1) facilitators of lifestyle modification, comprising perceived benefits and outcomes; social, family, and spiritual support; and medical advice and counselling; and (2) barriers to lifestyle modification, comprising lack of consistent motivation; psychological factors; physical limitations and health setbacks; social and environmental barriers; lack of guidance; misconceptions and beliefs; and financial constraints. Despite facing cultural, psychological, physical, and financial barriers, patients demonstrated willingness to adopt healthier lifestyles when supported by healthcare providers, family members, and personal motivation. These findings highlight the need for culturally sensitive, patient-centered interventions that integrate lifestyle counseling, psychosocial support, and community involvement to improve long-term cardiovascular outcomes.

## Introduction

Cardiovascular diseases (CVDs) encompass a range of conditions affecting the heart and blood vessels, such as coronary heart disease, stroke, and peripheral arterial disease [1]. Cardiovascular diseases represent a significant global health challenge, with an estimated 17.9 million deaths annually, accounting for about 32% of all global deaths, primarily due to heart attacks and strokes [2–4]. Alarmingly, over 75% of these deaths transpire in low- and middle-income countries (LMICs) [2,4]. If effective interventions are not put in place, CVD-related deaths are projected to increase to 23 million by 2030 [3,5]. Tanzania is among the countries facing a high burden of non-communicable diseases (NCDs); estimates indicate that NCDs account for approximately 33% of all deaths, while CVDs account for about 13% of all deaths [2–4].

Lifestyle modification is the cornerstone in helping CVD patients attain healthy lifestyle behaviors, with the potential benefits of promoting health and preventing complications [5]. Unhealthy lifestyles, including unhealthy diet, physical inactivity, smoking, and excessive alcohol consumption, are modifiable risk factors that contribute to the significant rise of CVD [6–8]. Addressing these risk factors through lifestyle modification interventions, including reduced salt intake, regular physical exercise for at least 150 minutes a week, maintaining a healthy weight, moderate alcohol consumption, and smoking cessation, is crucial for reducing the burden of CVDs [5,9–11]. Evidence shows that comprehensive lifestyle modification reduces CVD incidence, mortality, and readmission rates, emphasizing the importance of behavioral changes [7,12]. Non-compliance, conversely, increases the risk of severe CVDs, including stroke, heart failure, sudden death, and financial strain [6,13,14].

Among patients who already have CVD, lifestyle modification is used after diagnosis to reduce disease progression, complications, and recurrent cardiovascular events. In the classical public health prevention framework, the present study therefore concerns tertiary prevention, while many cardiovascular care guidelines describe similar measures within secondary-prevention care [9,10]. The relevant behaviors include reducing salt and unhealthy fats, increasing fruit and vegetable intake, engaging in regular physical activity appropriate to functional capacity, stopping smoking, and avoiding harmful alcohol use [5,9–11]. Importantly, the influences on these behaviors may differ: dietary change may depend on food affordability and household cooking practices, whereas physical activity may be constrained by symptoms and functional limitations [15,16]. Therefore, facilitators and barriers to lifestyle modification should be interpreted in relation to the specific personal, social, environmental, and health-system context in which each behavior occurs.

Despite lifestyle modification being one of the most cost-effective approaches to CVD management, low uptake of a healthier lifestyle persists [8]. A systematic review shows a low level of adherence to lifestyle modification (pooled mean of 27.4) among hypertensive patients, with a varying prevalence by region [17]. Studies across African countries have demonstrated suboptimal adherence to healthy lifestyle practices among patients at risk of CVD, with reported adherence levels ranging from 16.4% to 52.7% for CVDs [15,18–20]. Literature shows a vast number of personal, social, institutional, and cultural factors that influence CVD adherence to lifestyle modification among patients with CVD [17–19]. Health education has shown promise in motivating patients to adopt healthier lifestyles, yet adherence to recommended lifestyle modifications remains suboptimal [7,9,10]. A previous study conducted in Bangladesh reported experiences of lifestyle modification among patients with myocardial infarction; however, participants reported difficulty adjusting to the new life, including feeling stressed and living with fear [21].

So far in Tanzania, several measures to enhance lifestyle modifications have been deployed. These include infrastructure development, community health worker-based education, research-based interventions, health education and awareness campaigns, and government-led physical activity campaigns [1,22]. Despite these efforts, adherence to lifestyle modification among patients with CVD remains suboptimal, and many studies conducted in Tanzania and other African settings have primarily used quantitative approaches to estimate the prevalence and associated factors [1,15,18,19]. Although these studies provide important statistical insight, they offer a limited understanding of patients’ lived experiences, perceptions, and contextual challenges influencing adherence to lifestyle changes. Furthermore, findings from Tanzania indicate that, despite relatively good knowledge of NCD risk factors, unhealthy lifestyle practices continue to persist [3]. This knowledge–practice gap should not be interpreted as a simple failure of knowledge; behavior is shaped by interacting determinants including self-efficacy, motivation, social norms and support, environmental constraints, access to resources, and health-system influences. Qualitative inquiry is therefore needed to understand how these determinants operate in the Tanzanian context and influence patients’ ability to adopt and sustain lifestyle changes.

Furthermore, understanding patients’ own narratives can provide substantial insight into the real-life challenges and facilitators that shape adherence to a healthier lifestyle. Such context-specific understanding is essential for informing patient-centered interventions and improving healthcare delivery and outcomes. Therefore, this study aimed to explore perceived facilitators and barriers influencing lifestyle modification among adults with cardiovascular disease attending the cardiac clinic at Kilimanjaro Christian Medical Centre (KCMC), a referral hospital in Northern Tanzania.

## Materials and methods

### Ethics statement

Ethical approval for the study was obtained from the MUHAS Institutional Review Board (Ref. No. DA.282/298/01.C/2122). The potential participants were thoroughly informed about the study’s objectives, procedures, and their rights, and voluntary participation was encouraged before data collection. The first author ensured that participation did not interfere with clinic management services. To protect confidentiality and anonymity, interviews were conducted in a private room, minimizing environmental distractions and safeguarding participants’ privacy. Confidentiality was strictly upheld throughout the study by securing all personal information and restricting access to authorized personnel only. No identifying details were linked to the collected data, ensuring participants’ anonymity [23].

### Study design

This study employed an exploratory qualitative design to explore perceived facilitators and barriers influencing lifestyle modifications among adults with CVD. This design enabled the researchers to examine a topic with limited qualitative coverage in the Tanzanian context and allowed participants’ accounts to contribute to the development of context-specific knowledge [24].

### Study area

This study was conducted at Kilimanjaro Christian Medical Centre (KCMC), a zonal referral hospital in northern Tanzania located in Kilimanjaro region. Serving five regions: Kilimanjaro, Arusha, Manyara, Tanga, and Singida, along with parts of Kenya, KCMC provides specialized cardiovascular services for a wide range of conditions, including myocardial infarction, coronary artery disease, and rheumatic heart disease. Its role as a referral center makes it an ideal setting for exploring influences on lifestyle modification among adults with cardiovascular diseases in the region.

### Study population and eligibility criteria

The study population included adults with CVD attending the outpatient cardiac clinic at KCMC. Inclusion criteria were age 18 years or older, a CVD diagnosis for at least six months, ability to communicate in Swahili and willingness to provide written informed consent. Patients who were acutely ill or experiencing mental health challenges that could interfere with participation were excluded.

### Sample size and sampling procedure

A total of ten (10) participants were purposively recruited from the outpatient cardiac clinic. Sampling sought variation in age, sex, and type of cardiovascular disease to capture a range of experiences. Recruitment and data collection occurred concurrently. The final sample size was guided by thematic saturation rather than statistical representation [25]. Saturation was reached after eight (8) interviews, and two (2) additional participants were interviewed to confirm that no new codes, themes, or substantive insights emerged.

### Data collection method and tool

Data were collected using a semi-structured interview guide developed to explore participants’ perceived influences on lifestyle modification among adults with CVD (S1 Text). The guide included open-ended questions on support systems and resources, motivation for lifestyle change, guidance from healthcare professionals, challenges in sustaining changes over time, and coping with setbacks or obstacles. Follow-up probes were used to clarify responses and explore emerging issues. Because the guide examined lifestyle modification broadly rather than systematically assessing each behavior separately, the findings are interpreted as participants’ perceived influences on lifestyle modification rather than as a measure of specific lifestyle practices.

### Data collection procedure

After obtaining the necessary permissions, the researcher was introduced to the person in charge of the cardiac clinic, who facilitated access to potential participants. Potential participants were informed about the study purpose, benefits, risks, and voluntary nature of participation, and written informed consent was obtained before interviews. The study was conducted between May and July 2024. Interviews were conducted in Swahili in a private room at the cardiac clinic to ensure confidentiality and accommodate clinic schedules. We acknowledge that data were collected by trained nurses from different nursing specialty backgrounds, including those with teaching and clinical experience in cardiovascular and chronic disease care in Tanzania. This positionality provided contextual understanding of the subject matter but could also introduce pre-existing assumptions about patients’ knowledge, behaviors, and adherence to lifestyle recommendations. To address this, researchers maintained reflective field notes after interviews, documenting personal assumptions, reactions, and emerging interpretations. Non-judgmental, open-ended questioning was used to encourage participants to express their views freely. Each interview was audio-recorded and lasted approximately 20–30 minutes. The focused open-ended guide and follow-up probing allowed participants to provide detailed accounts within this period while minimizing participant fatigue and accommodating clinic schedules. Data adequacy was judged by thematic saturation rather than interview duration.

### Data analysis

Data were analyzed manually using Braun and Clarke’s six-phase thematic analysis [26]. To support reflexive analysis, the research team discussed how professional and cultural backgrounds could shape interpretation, and coding was conducted inductively so that codes and themes were grounded in participants’ narratives rather than predetermined frameworks. First, GNM and BM repeatedly read the interview transcripts to become familiar with the data and identify preliminary patterns. Second, audio recordings were transcribed and translated from Swahili to English by GNM, after which BM cross-checked selected transcripts against the audio recordings to support accuracy and consistency of translation. GNM and BM then independently generated initial codes by identifying features relevant to lifestyle modification, compared their coding, and discussed discrepancies. Third, related codes were organized into potential themes. Fourth, preliminary themes were reviewed and refined with NM, ET, and MN, who are experienced qualitative researchers, to ensure that they reflected both the coded extracts and the overall dataset. Fifth, the research team collaboratively defined and named each theme. Finally, the themes were developed into a coherent analytical narrative supported by illustrative participant quotations. These steps enhanced the transparency, credibility, and confirmability of the analysis.

### Trustworthiness of data

Rigor of the study findings was ensured by using an explanation of four established principles of Guba and Lincoln for qualitative trustworthiness criteria of credibility, dependability, confirmability, and transferability. Credibility was ensured through persistent observation, extended field time, well-prepared interview guides, and virtual meetings with supervisors. Probe questions, iterative questioning, and paraphrasing of responses were employed to obtain detailed information. Initial findings were shared with informants during hospital visits to ensure their perspectives were considered.

Data were transcribed verbatim from recorded files for analysis. Dependability was ensured through meticulous documentation, with informants selected purposefully. Interviews were triangulated through observation and field notes, and moderation was employed. Transcripts, field notes, and audio recordings were used to document the data collection. Confirmability was ensured by monitoring the recorded data process, triangulation of data, and double-checking. Quotes from participants supported the study findings. Transferability was ensured by providing a detailed description of the study setting and informants’ characteristics, enabling comparisons to similar situations in Tanzania and other countries.

## Results

The study included ten participants with ages ranging from 49 to 68 years. The duration since cardiovascular disease diagnosis ranged from six months to fourteen years. Some participants had co-morbid conditions like hypertension and diabetes mellitus, as shown in Table 1.

**Table 1:** Sociodemographic characteristics of the study participants (n = 10).

Participant No.	Age	Sex	Education Level	Occupation	Time Since CVD, HTN & Comorbid conditions
1	52	Male	Primary Education	Businessman	1 year CVD,5years, DM,3years HTN
2	58	Female	Primary Education	Housewife	4 years CVD
3	50	Female	College	Teacher	3 years CVD
4	60	Male	Primary Education	Businessman and Farmer	3 years CVD, 12 years HTN
5	68	Female	Primary Education	Housewife	8 years CVD
6	50	Female	Primary Education	Farmer	5 years CVD
7	59	Female	Primary Education	Farmer	>4 years CVD
8	63	Male	College	Retired Medical recorder officer	6 months CVD, 10 years DM + HTN
9	62	Male	Secondary Education	Farmer	10 years CVD + HTN
10	49	Female	Primary Education	Farmer	14 years CVD + HTN

The results generated two main themes: (1) facilitators of lifestyle modification, with three sub-themes: perceived benefits and outcomes; social, family, and spiritual support; and medical advice and counselling; and (2) barriers to lifestyle modification, with seven sub-themes: lack of consistent motivation; psychological factors; physical limitations and health setbacks; social and environmental barriers; lack of guidance; misconceptions and beliefs; and financial constraints. The themes and associated sub-themes are summarized in Table 2 below.

**Table 2:** Emerging themes and sub-themes on facilitators and barriers to lifestyle modification among adults with cardiovascular diseases attending clinic at Kilimanjaro Christian Medical Centre.

Themes	Sub-themes
1. Facilitators of lifestyle modification	i) Perceived benefits and outcomes
	ii) Social, family, and spiritual support
	iii) Medical advice and counselling
2) Barriers to lifestyle modification practices	i) Lack of consistent motivation
	ii) Psychological factors
	iii) Physical limitations and health setbacks
	iv) Social and environmental barriers
	v) Lack of guidance
	vi) Misconceptions and beliefs
	vii) Financial constraints

### Theme 1: Facilitators of lifestyle modification practices

#### Perceived benefits and outcomes of lifestyle modifications.

Patients’ perceptions of the benefits and outcomes of lifestyle modifications played a crucial role in shaping their commitment to change. Those who experienced noticeable improvements in their health, such as feeling healthy or reduced symptoms, felt encouraged to maintain their modified routines.

*“I started feeling more energetic and less out of breath after sticking to my new routine for a few months. Seeing those changes motivated me to keep going.”* (P8, 6 months with CVD)

*“The motivation comes from seeing improvements in their health and knowing that other patients who follow these lifestyles changes continue living and supporting their families.”* (P3, 3 years with CVD)

Some participants attributed improvements in their health to the lifestyle changes they had. Lowering blood sugar levels and normalizing blood pressure were noted as direct outcomes of their dietary adjustments and increased physical activity.

This improvement not only enhanced their physical well-being but also contributed to a reduction in fatigue, enabling them to engage more actively in daily activities with renewed energy and vigor.

*“I do it because, when I exercise, my blood sugar drops from high levels to seven, eight, six” (*P1, 1 year with CVD*).*

*“Yes! They improve my health” (*P3, 3 years with CVD*).*

*“After treatment, I knew I had to follow the guidance. Seeing others enjoy themselves while I couldn’t, but I had to manage.”* (P4, 3 years with CVD)

Across these accounts, visible improvements in symptoms and physiological measures reinforced participants’ belief that lifestyle changes were worthwhile, strengthening motivation to continue.

#### Social, family, and spiritual support.

Beyond individual efforts, the pivotal role of family support emerged prominently in facilitating participants’ adherence to lifestyle modifications. Participants highlighted the integral support provided by children and spouses, who not only assisted in practical ways like meal preparation but also offered emotional encouragement and financial help. This familial involvement was crucial in sustaining motivation and reinforcing the importance of healthy habits in daily life.

*“When I cook in one pot, I don’t add salt. They tolerate this, and later they add salt to their portion, but the food doesn’t taste the same.”* (P3, 3 years with CVD)

*“My children, especially the girls, help a lot” (*P4, 3 years with CVD*).*

*“My spouse has been very supportive, especially in preparing healthy meals” (*P7, 4 years with CVD*).*

Spiritual beliefs also functioned as a source of emotional support. Faith helped some participants manage stress, remain committed to treatment, and resist advice to replace hospital care with traditional healing practices.

*“I’ve prayed to God to help me reduce my stress so I can manage my condition and take my medication. I don’t have any more worries or stress”* (P6, 5 years with CVD).

*“…people have advised me to see traditional healers because of my leg pain (oedema), saying it’s not just physical …, but I’ve told them I’m not ready for that. I believe only God can help me, so I stick to going to the hospital and praying.”* (P7, 4 years with CVD)

These accounts show that support was not limited to practical help with meals; family relationships and spiritual beliefs also provided emotional reinforcement that helped participants sustain lifestyle change.

#### Medical advice and counselling.

Participants emphasized the crucial role of medical advice and regular clinic attendance in managing their cardiovascular health. Adhering to prescribed treatment plans and maintaining consistent medical consultations were regarded as essential components for achieving and sustaining health goals. This structured approach not only offered participants personalized guidance but also fostered a sense of accountability in upholding their health regimen. Participants explained:

*“I never miss my clinic appointments because the doctors help me stay on track. When they explain things clearly, I understand what to do and feel more confident about my health”* (P6, 5 years with CVD).

*“Regular visits to the clinic have kept me on track with my health goals”* (P7, 4 years with CVD).

Healthcare guidance also acted as a source of motivation. Participants described dietary and alcohol-related advice from clinicians as credible direction for decisions they had to make outside the clinic.

*“When you visit a doctor, they ask if you use a lot of salt. If you say you don’t know because you’re not the cook, they advise you to tell the cook to use less salt, and they also advise against drinking a lot of alcohol”* (P8, 6 months with CVD).

*“When you come to the clinic, they explain through the doctors, and you decide to follow the advice they give you”* (P3, 3 years with CVD).

Together, these narratives indicate that repeated, understandable clinical advice could strengthen confidence and motivation, although the amount of guidance received varied across participants.

### Theme 2: Barriers to lifestyle modification practices

Seven (7) sub-themes emerged under this theme: lack of consistent motivation, psychological factors, physical limitations and health setbacks, social and environmental barriers, lack of guidance, misconceptions and beliefs, and financial constraints.

#### Lack of consistent motivation.

Many cardiovascular patients report initial enthusiasm for lifestyle changes but struggle to maintain motivation over time. The demands of daily life and the absence of immediate health improvements contribute to waning commitment.

*“At first, I was very dedicated to following the diet and exercise plan, but after a few months, it became really hard to keep up. I didn’t see any quick results, and that made it even harder to stay motivated.”* (P3, 3 years with CVD)

*“I don’t think so because if reducing salt and oils had benefits, my condition would have either gone away or improved significantly. But it keeps recurring.”* (P7, 4 years with CVD)

These accounts suggest that motivation was closely tied to whether participants perceived noticeable benefits, so recurrence of symptoms could weaken confidence in recommended changes.

#### Psychological factors.

Anxiety, depression, and stress was reported negatively to impact patients’ ability to maintain lifestyle changes. These psychological factors were described to diminish their capacity to stay consistent with modifications such as diet and exercise.

*“When I feel stressed or down, I tend to eat junk food and skip workouts. It’s like a vicious cycle because I know I need to make these changes for my health, but my mental state makes it so difficult.”* (P8, 6 months with CVD)

Stress therefore functioned not only as an emotional burden but also as a behavioral trigger that disrupted eating and activity routines.

#### Physical limitations and health setbacks.

Some patients found it difficult to maintain physical activity due to existing health conditions or setbacks related to their cardiovascular issues. Pain, fatigue, and other symptoms can interfere with regular exercise routines.

*“I try to walk every day, but some days the pain in my legs is just too much. There are times when I feel too tired to do anything, and that makes it hard to keep up with my exercise routine.”* (P7, 4 years with CVD)

Some participants reported medication side effects and health issues, like swelling and chest tightness, posed significant barriers to maintaining recommended lifestyle changes. They described how these challenges restricted their ability to engage in physical activities or adhere to dietary restrictions, thereby impacting their overall health outcomes. They said:

*“The medication caused changes in my body, like blurred vision and increased body heat” (*P2, 4 years with CVD*).*

*“I do, my child. First, I try to walk long distances, but I find it difficult as I get out of breath and need to use a walking stick.”* (P6, 5 years with CVD)

*“I used to exercise, but recently I had to stop due to swelling of the legs and chest tightness” (*P8, 6 months with CVD*).*

Physical symptoms and perceived medication effects reduced participants’ capability to remain active, illustrating that recommendations for exercise were not always feasible without clinical adaptation.

#### Social and environmental barriers.

The participants reported facing social pressures and environmental constraints that hinder their ability to sustain lifestyle changes. Factors such as family habits, lack of support, and the availability of unhealthy food options play significant roles.

*“My family loves fried food, and it’s always around. Even when I try to stick to healthier options, it’s tough because I feel left out during meals.”* (P4, 3years with CVD)

*“Honestly, changes are good but hard to follow, because I don’t cook. I live with my daughter-in-law and her child, who usually cooks. If I tell her to reduce salt, she might say, “Sorry, Grandma, I added too much.”* (P5, 8 years with CVD)

These experiences show that dietary change was negotiated within shared households; when participants did not control food preparation, individual intentions could be overridden by family routines.

#### Lack of guidance.

Experiences of healthcare guidance were mixed. While some participants described useful advice during clinic visits, others reported limited information or follow-up on how to sustain lifestyle changes. Under this sub-theme, participants emphasized the occasions when guidance was absent, insufficient, or not reinforced over time.

*“No, I have never received any [information about lifestyle modification]” (*P1, 1 year with CVD*).*

*“No, it’s just me; the nurses and doctors don’t follow up closely about my lifestyle changes.”* (P9, 10 years with CVD)

These narratives suggest that the barrier was not necessarily a complete absence of counselling, but inconsistency in the depth, continuity, and follow-up of lifestyle support across clinic encounters.

#### Misconceptions and beliefs about lifestyle modification.

Superstitions, traditional beliefs, and misconceptions about health and lifestyle changes were identified as potential barriers. Participants discussed how cultural beliefs influenced their perception of illness and treatment, sometimes conflicting with medical advice. Overcoming these deeply ingrained beliefs proved challenging, as community misconceptions continued to influence their health-related decisions.

*“They tell you superstitions, to go there, do this, but you see that superstition can’t settle in your heart*. *When you have undergone tests that show this and that, you should follow the health professionals.” (*P1, 1 year with CVD*).*

*“Many people in my community still don’t understand the importance of these lifestyle changes” (*P7, 4 years with CVD*).*

*“At first, I tried traditional methods before coming to the hospital, but I realized I needed proper treatment.”* (P9, 10 years with CVD)

Participants therefore navigated competing explanations of illness, and trust in biomedical testing and hospital care appeared to help some resist community pressure toward alternative explanations or treatments.

#### Financial constraints.

The cost of healthier food options, and other lifestyle modification resources can be prohibitive for some patients, leading to lapses in their adherence to recommended changes.

*“I know I should buy fresh vegetables and lean meats, but they are expensive. I can’t always afford them, so I end up buying cheaper, less healthy options. Going to the gym is also out of my budget.”* (P6, 5 years with CVD)

*“Yes, it depends on the resources available; sometimes you eat (leafy greens and fruits), sometimes you don’t, because of life’s challenges, sometimes you can’t follow through.”* (P10, 14 years with CVD)

Financial limitations narrowed the range of feasible choices, particularly for diet and paid exercise options, meaning that recommended changes could be difficult to sustain even when participants understood and valued them.

## Discussion

This study shows that lifestyle modification among adults living with cardiovascular disease (CVD) was influenced by interacting personal, interpersonal, clinical, social, and economic factors rather than knowledge alone. Participants were more likely to sustain recommended changes when they experienced tangible health improvements, received family support, drew on spiritual beliefs, and obtained understandable healthcare guidance. In contrast, psychological stress, physical symptoms, household practices, inconsistent counselling, misconceptions, and financial constraints hindered adherence. These findings are broadly consistent with studies from Tanzania and Ethiopia showing that knowledge, self-efficacy, social support, and patient–provider relationships influence lifestyle modification [18,19]. However, while Shilole et al. found that adequate knowledge was associated with better adherence [19], the present findings show that knowledge alone may not translate into practice when patients face physical, social, or financial constraints. This difference may reflect the qualitative design of the present study, which captured how patients negotiate these barriers in daily life. Cardiovascular care should therefore assess not only what patients know, but also whether they have the practical and social conditions needed to act on that knowledge.

Social cognitive theory provides a useful post hoc lens for interpreting the role of perceived benefits, self-efficacy, social reinforcement, and environmental influences observed in this study [27]. The theory was not used prospectively to structure the interview guide, but its concepts help explain how participants’ confidence, perceived health improvements, and social support influenced lifestyle modification. The findings can also be understood through the Capability, Opportunity, Motivation-Behaviour Model (COM-B model), whereby capability was influenced by symptoms and understanding, opportunity by family practices, healthcare support, and affordability, and motivation by perceived benefits, stress, beliefs, and persistence [28]. Importantly, these findings emphasize that information and knowledge alone are insufficient to produce or sustain lifestyle behaviour change. Although education can improve patients’ understanding of recommended lifestyle modifications, translating this knowledge into practice requires additional support that addresses motivation, physical capability, social and family influences, financial constraints, and opportunities within the patient’s environment. This interpretation extends previous studies highlighting the role of education and knowledge in lifestyle modification [18,19] by demonstrating that knowledge must be accompanied by interventions that address the broader behavioral and contextual determinants of change. In contrast, research from Dutch cardiac care identified eHealth and digital monitoring as important facilitators [29], suggesting that the type of supportive intervention may vary according to healthcare resources and context. Future interventions in this setting should therefore address capability, opportunity, and motivation together rather than relying primarily on information provision.

Perceived health improvements, family involvement, spirituality, and healthcare guidance were important facilitators because they made lifestyle recommendations meaningful within daily life. Participants described improved energy, reduced breathlessness, lower blood glucose, and normalized blood pressure, which appeared to reinforce continued engagement. Similar findings have been reported among patients with chronic heart failure and other cardiovascular conditions, where family and healthcare support facilitated self-care and healthy behaviour [8,13]. However, Saavedra Espinosa et al. reported improvement following structured face-to-face and telephone follow-up [8], whereas participants in the present study described inconsistent guidance and limited follow-up. This difference may reflect the structured nature of the intervention compared with routine clinic-based counselling. Referral cardiac clinics may therefore benefit from repeated, family-inclusive and behaviour-specific counselling that reinforces observed health improvements and incorporates culturally relevant sources of support.

The barriers identified further show that lifestyle modification should not be treated as a single behaviour. Physical activity was limited by breathlessness, pain, swelling, fatigue, and reduced mobility, while dietary modification was affected by household food preparation, family preferences, and affordability. Psychological stress and fluctuating motivation also undermined sustained change. These findings are similar to reports from Bangladesh and Denmark, where stress, fear, financial constraints, and social influences affected lifestyle adjustment [21,30]. However, Nielsen et al. identified lack of time as the most common exercise barrier [30], whereas participants in the present study more often described symptoms, functional limitations, household dependence, and financial difficulties. This difference may reflect that the Danish study involved adults considering preventive lifestyle changes, whereas participants in the present study were already living with established CVD. Counselling should therefore address each behaviour separately and prioritize affordable dietary options, symptom-adapted physical activity, family involvement, and psychosocial support.

The principal contribution of this study is therefore not simply the identification of another list of facilitators and barriers, but a context-specific explanation of how these factors interact among adults already living with CVD. Previous studies have emphasized social support, health education, motivation, and environmental influences [8,13,18], while the present study highlights how these factors may be reinforced or undermined by household decision-making, illness-related limitations, financial constraints, spiritual coping, and continuity of counselling. In contrast to settings where digital or highly structured rehabilitation support is more available [29], feasible interventions in this context may need to prioritize repeated face-to-face counselling, family participation, affordable local dietary strategies, symptom-adapted activity, and motivational and psychosocial support. Future studies should use behaviour-specific, theory-informed, and pretested tools and evaluate multi-component interventions addressing capability, opportunity, and motivation [28].

### Strengths of the study

This study demonstrates several key strengths that contribute to its credibility and relevance. Conducted at KCMC, a major referral center in Northern Tanzania, it captured diverse perspectives from cardiovascular patients within a real-world clinical setting. The use of in-depth, semi-structured interviews allowed participants to express their experiences openly, resulting in rich, nuanced data. Conducting interviews in Swahili further enhanced the authenticity and clarity of responses, making the findings culturally and linguistically appropriate.

The application of Braun and Clarke’s inductive thematic analysis ensured a systematic and grounded approach to identifying themes, allowing insights to emerge directly from participant narratives. Ethical considerations were strongly upheld, with informed consent, privacy, and confidentiality maintained throughout the process. Additionally, determining the sample size based on data saturation ensured that the data collected was both comprehensive and thematically complete.

### Limitations of the study

This study has several limitations. First, participants were recruited from a single referral hospital and only from patients attending follow-up care, which may introduce selection bias because clinic attendees may be more engaged with cardiovascular care than patients who miss appointments or receive care elsewhere. The sample of ten participants was appropriate for the depth-oriented qualitative design and saturation achieved in this setting, but it limits transferability to other hospitals, primary-care settings, and patient groups. Second, findings were based on self-reported retrospective accounts and may therefore be affected by social desirability and recall bias. Third, the nature of the interviews captured experiences at one point in time and cannot establish how facilitators, barriers, or behaviors change over time or establish causal relationships. Fourth, translation from Swahili to English may have reduced some linguistic nuance. Finally, the researchers’ nursing and clinical backgrounds could have influenced data collection and interpretation; reflective field notes, independent coding, and team-based theme review were used to reduce this influence.

## Conclusion and recommendations

This study shows that lifestyle modification among adults with established cardiovascular disease is shaped by the interaction of perceived health benefits, family and spiritual support, healthcare guidance, motivation, psychological wellbeing, physical capability, household and community influences, and financial resources. For clinical practice, cardiac services should move from general one-time advice toward repeated, behavior-specific counselling that considers functional limitations, affordability, psychological distress, and the patient’s household context, with family involvement where appropriate. Future interventions should be theory-informed and designed around the specific capability, opportunity, and motivational barriers identified for diet, physical activity, tobacco use, and alcohol use. Multi-site qualitative, longitudinal, and mixed-methods studies are needed to assess how these influences vary across settings and to test feasible, culturally appropriate strategies for sustaining cardiovascular risk-reduction behaviors over time.

## Supporting information

S1 Text Semi-structured interview guide. The interview guide used to explore perceived facilitators and barriers influencing lifestyle modification among adults with cardiovascular disease attending the cardiac clinic at Kilimanjaro Christian Medical Centre, Tanzania.
(DOCX)
