# Supplementary material for: Perceived facilitators and barriers influencing lifestyle modification among adults with cardiovascular disease attending a cardiac clinic at a referral hospital in northern Tanzania: An exploratory qualitative study
Source: PLOS Glob Public Health. 2026 Oct 27;6(10):e0007537. doi: 10.1371/journal.pgph.0007537 (PMC13712588; doi:10.1371/journal.pgph.0007537)
Supplement: Supplementary file 1 [file pgph.0007537.s001.docx]

**IDI GUIDE (ENGLISH VERSION)**

**Dear Participant,**

Welcome to our qualitative study on "Facilitators and Barriers to Lifestyle Modification Practices Among Cardiovascular Patients Attending Cardiac Clinic at Kilimanjaro Christian Medical Centre." Your participation in this research is invaluable as we aim to gain deeper insights into the factors influencing lifestyle modifications among individuals living with cardiovascular diseases (CVD).

**Purpose of the Study:**

This study aims to explore the various lifestyle modification practices adopted by cardiovascular patients, identify the facilitators that support these practices, and understand the barriers that hinder effective lifestyle modifications among this population. By shedding light on these aspects, we aim to inform healthcare providers and policymakers about potential strategies to enhance lifestyle interventions for better cardiovascular health outcomes.

**Guided Questions:**

**Domain I: Lifestyle Modification Practices Among CVD Patients**

1. Can you describe the lifestyle modifications you have adopted since being diagnosed with cardiovascular disease?

2. How do you perceive the role of lifestyle changes in managing your cardiovascular condition?

3. What factors influence your adherence to these lifestyle modifications?

4. Can you share any challenges or successes you've experienced in implementing these changes into your daily life?

**Domain II: Facilitators to Lifestyle Modifications Practices Among CVD Patients**

1. Could you discuss any external support systems or resources that have facilitated your adherence to lifestyle modifications?

2. In your opinion, what motivates individuals with cardiovascular diseases to engage in positive lifestyle changes?

3. Have you received any guidance or encouragement from healthcare professionals regarding lifestyle modifications?

4. Can you describe any instances where social or familial support has helped you maintain your lifestyle changes?

**Domain III: Barriers to Lifestyle Modifications Practices Among CVD Patients**

1. What are some of the challenges you face in sustaining your lifestyle modifications over time?

2. Are there any environmental or societal factors that make it difficult for you to adhere to your prescribed lifestyle changes?

3. How do you cope with setbacks or obstacles in maintaining your lifestyle modifications?

4. Have you encountered any misconceptions or beliefs that hinder your commitment to lifestyle changes?

These questions are designed to encourage open and honest discussions about your experiences with lifestyle modifications as a cardiovascular patient. Your insights will contribute significantly to our understanding of this important aspect of cardiovascular care. We sincerely appreciate your time and participation in this study.

Thank you.

**IDI GUIDE (KISWAHILI VERSION)**

**Mpendwa Mshiriki**,

Karibu katika utafiti wetu wa kina kuhusu "Wahamasishaji na Vipingamizi vya Mabadiliko ya Mwenendo wa Maisha Miongoni mwa Wagonjwa wa Moyo Wanaohudhuria Kliniki ya Moyo katika Kituo cha Matibabu cha Kikristo cha Kilimanjaro." Ushiriki wako katika utafiti huu ni muhimu kwani lengo letu ni kupata ufahamu zaidi kuhusu mambo yanayoathiri mabadiliko ya maisha kati ya watu wanaoishi na magonjwa ya moyo.

**Lengo la Utafiti**:

Lengo la utafiti huu ni kuchunguza mazoea mbalimbali ya mabadiliko ya maisha yanayofuatwa na wagonjwa wa moyo, kutambua wahamasishaji wanaounga mkono mazoea haya, na kuelewa vizuizi vinavyokwamisha mabadiliko yenye ufanisi ya mtindo wa maisha kati ya kundi hili la watu. Kwa kufichua mwangaza juu ya vipengele hivi, lengo letu ni kuwaelimisha watoa huduma za afya na watunga sera kuhusu mikakati inayowezekana ya kuboresha marekebisho ya mtindo wa maisha kwa matokeo bora ya afya ya moyo.

Maswali ya Mwongozo:

**Sehemu I**: Mabadiliko ya Mwenendo wa Maisha Kati ya Wagonjwa wa Moyo

1. Je, unaweza kuelezea mabadiliko ya maisha uliyoyachukua tangu kugunduliwa na ugonjwa wa moyo?

2. Unachukuliaje jukumu la mabadiliko ya mtindo wa maisha katika kusimamia hali yako ya moyo?

3. Ni mambo gani yanayowaathiri katika kuzingatia mabadiliko haya ya mtindo wa maisha?

4. Je, unaweza kushiriki changamoto au mafanikio uliyopata katika kutekeleza mabadiliko haya katika maisha yako ya kila siku?

**Sehemu II**: Wahamasishaji wa Mabadiliko ya Mwenendo wa Maisha Kati ya Wagonjwa wa Moyo

1. Je, unaweza kujadili mifumo au rasilimali za nje ambazo zimekuwezesha kuzingatia mabadiliko ya mtindo wa maisha?

2. Kwa maoni yako, ni nini kinachowatia moyo watu wenye magonjwa ya moyo kufanya mabadiliko chanya katika mtindo wao wa maisha?

3. Je, umepokea mwongozo au kushauriwa na wataalamu wa afya kuhusu mabadiliko ya mtindo wa maisha?

4. Je, unaweza kuelezea hali yoyote ambapo msaada wa kijamii au wa kifamilia umekusaidia kudumisha mabadiliko ya mtindo wa maisha?

**Sehemu III**: Vipingamizi vya Mabadiliko ya Mwenendo wa Maisha Kati ya Wagonjwa wa Moyo

1. Ni changamoto gani unakabiliana nazo katika kudumisha mabadiliko ya mtindo wa maisha kwa muda?

2. Je, kuna sababu za mazingira au kijamii zinazofanya iwe ngumu kwako kuzingatia mabadiliko ya mtindo wa maisha uliopendekezwa?

3. Unawezaje kukabiliana na vikwazo au vizuizi katika kudumisha mabadiliko ya mtindo wa maisha?

4. Umekumbana na dhana potofu au imani zinazokwamisha azma yako ya kufanya mabadiliko ya mtindo wa maisha?

Maswali haya yameundwa kukuhamasisha kufanya majadiliano wazi na ya kweli kuhusu uzoefu wako na mabadiliko ya mtindo wa maisha kama mgonjwa wa moyo. Mawazo yako yatachangia kwa kiasi kikubwa katika ufahamu wetu wa sehemu muhimu hii ya huduma ya moyo. Tunathamini sana muda wako na ushiriki katika utafiti huu.
