# Supplementary material for: Perceived facilitators and barriers influencing lifestyle modification among adults with cardiovascular disease attending a cardiac clinic at a referral hospital in northern Tanzania: An exploratory qualitative study
Source: PLOS Glob Public Health. 2026 Oct 27;6(10):e0007537. doi: 10.1371/journal.pgph.0007537 (PMC13712588; doi:10.1371/journal.pgph.0007537)
Supplement: Supplementary file 2 [file pgph.0007537.s003.docx]

**Rebuttal Letter**

**Manuscript ID:** PGPH-D-25-02058

**Title:** *Facilitators and Barriers to Lifestyle Modification Practices among Cardiovascular Patients attending Cardiac Clinic at a Faith-Based Hospital in Northern Tanzania*
**Journal:** *PLOS Global Public Health*

**Dear Editor,**

We sincerely thank the handling editor and reviewer for their constructive and insightful comments on our manuscript. We have carefully revised the paper in response to all suggestions and believe that these improvements have significantly strengthened its clarity, methodological rigor, and contextual relevance. Below is a detailed, point-by-point response outlining how each issue has been addressed.

**1. Competing Interest Statement**

**Editorial Comment:**

We have amended your Competing Interest statement to comply with journal's style. Please confirm its accuracy.

**Response:**
We have reviewed the amended Competing Interest statement and confirm that it is correct. The authors declare that there are no competing interests.

**2. Financial Disclosure Statement**

**Editorial Comment:**

Please provide a detailed financial disclosure statement in full sentences using the exact wording you wish to publish.

**Response:**
We have now included the following statement in the manuscript:

**Financial Disclosure:** This study was funded by the Government of Tanzania through the Ministry of Health. However, the funder had no role in study design, data collection and analysis, decision to publish, or preparation of the manuscript. All data are available without restrictions.

**3. Data Availability Statement**

**Editorial Comment:**

Please clarify the nature of restrictions on sharing de-identified data, who imposed them, and how access can be requested.

**Response:**
All relevant data are within this paper

**4. Reviewer #1 Comments and Our Responses**

**Introduction**

**Comment:** The introduction provides good context but is slightly repetitive and could better highlight the gap; add justification for using qualitative methods in LMICs.

**Response:**
We have refined the introduction to remove repetition, especially for cardiovascular disease statistics, and added justification for the qualitative approach, emphasizing its value in understanding contextual and cultural influences on patient behavior in low- and middle-income countries.

**Methods**

a) **Sampling:** Reviewer noted that *n = 10* is small but justified by saturation; requested more information on recruitment diversity.

**Response:**
We have clarified that participants were purposively selected to ensure diversity in gender, age, and cardiovascular conditions to capture a wide range of perspectives.

**b) Data Collection (Interview Duration):** Reviewer stated that 20–30 minutes may be short for in-depth interviews.

**Response:**
We added the following justification in the *Data Collection* subsection:

“Although interviews lasted approximately 20–30 minutes, they were sufficiently comprehensive to capture in-depth experiences due to the focused nature of the guide and participants’ familiarity with the study context. Data saturation was achieved within this timeframe, which aligns with similar qualitative studies conducted in clinical settings where patients have limited availability.”

**c) Reflexivity and Rigor:** Reviewer requested information on pilot testing, researcher reflexivity, and analytical rigor.

**Response:**
We have indicated that the interview guide was piloted with two participants to ensure clarity. Additionally, we described researcher reflexivity, noting that data collection and analysis were conducted by trained nurse researchers aware of potential biases, and themes were reviewed by supervisors to ensure credibility.

**d) Analysis:** Reviewer asked for details on software and inter-rater reliability.
**Response:**
We added that qualitative data were analyzed manually through thematic analysis guided by Braun and Clarke’s framework, with coding cross-checked by two independent reviewers to ensure consistency and reliability.

**e) Study Period:** Reviewer noted inconsistency between abstract and text.

**Response:**
The study period has been harmonized to read consistently as *May–July 2024* throughout the manuscript.

**Results and Discussion**

**Comment:** Reviewer suggested improving thematic structure, deepening discussion synthesis, and emphasizing local context and novelty.

**Response:**
We reorganized the results to present barriers and facilitators clearly under distinct thematic categories. Although this study was conducted at a faith-based hospital, the institution provides care following standard medical protocols, and participants reported that their lifestyle modification experiences were guided by healthcare advice and social support rather than religious teachings. Hence, the faith-based nature of the hospital did not appear to influence patients’ adherence to lifestyle modification recommendations.

**Conclusion**

**Comment:** Reviewer noted that the conclusion was repetitive and lacked implications for policy and practice.

**Response:**
We have shortened and refined the conclusion to summarize the study succinctly and added explicit implications for practice and policy, as follows:

“This study highlights the critical role of healthcare providers, particularly cardiovascular nurses, in delivering patient-centered education and psychosocial support to promote sustainable lifestyle changes. Findings call for policy emphasis on strengthening lifestyle counseling, integrating culturally sensitive health education, and enhancing follow-up systems within cardiac care programs to improve long-term patient outcomes.”

**Language and Style**

**Comment:** Reviewer recommended proofreading and correcting grammatical errors.
**Response:**
The manuscript has been thoroughly proofread and edited for grammatical accuracy, clarity, and coherence.

**Summary**

All requested corrections and additions have been incorporated into the revised manuscript, including updates to:

- Competing Interest statement
- Financial Disclosure
- Data Availability statement
- Methods section (sampling, data collection, rigor, and analysis)
- Discussion and Conclusion (depth, synthesis, implications)

We are grateful for the thoughtful feedback, which has substantially improved the quality and presentation of our manuscript. We hope the revised version now meets the publication standards of *PLOS Global Public Health.*

**Sincerely,**
**Benedicto Tenson Mgala**

Corresponding Author

Department of Clinical Nursing

Muhimbili University of Health and Allied Sciences (MUHAS)

Dar es Salaam, Tanzania

Email: [benedictomgala@gmail.com](mailto:benedictomgala@gmail.com)
