# Supplementary material for: Perceived facilitators and barriers influencing lifestyle modification among adults with cardiovascular disease attending a cardiac clinic at a referral hospital in northern Tanzania: An exploratory qualitative study
Source: PLOS Glob Public Health. 2026 Oct 27;6(10):e0007537. doi: 10.1371/journal.pgph.0007537 (PMC13712588; doi:10.1371/journal.pgph.0007537)
Supplement: Supplementary file 3 [file pgph.0007537.s005.docx]

**Comments**

**Title:**

The title is too long consider removing faith-based unless it is central to analysis

**Abstract and Keywords:**

- There grammar issues e.g. where you state “there are limited research exploring lifestyle modification practices…”
- The abstract is concise but could more explicitly state the sample size (n=10) and key sub-themes for facilitators and barriers. The conclusion is vague. Consider revising to show clearer implications of study.
- Keywords are appropriate (cardiovascular disease; Lifestyle Modification; Behavioral Change") but add "Tanzania" or "qualitative study" for better indexing.

**Introduction:**

- A strong global and local context has been highlighted (e.g., 13% of NCD deaths in Tanzania attributed to CVDs). However, fix minor formatting inconsistencies in citations, e.g., “(2).(3,4)” on page 5 should be “(2–4)” for consistency. Several paragraphs repeat the same ideas e.g when you talk about CVD burden.
- There are grammar issues for instance when you say… “Studies conducted in African countries show that the adherence to a healthier lifestyle for patients at risk of CVDs ranges between 16.4%-52.7%.” Consider rephrasing.
- The gap analysis is unclear (e.g., quantitative studies exist, but qualitative narratives are lacking). Consider highlighting what other studies have/whet they missed and why the qualitative evidence is needed.

**Materials and Methods:**

- The section is detailed and transparent. The addition of reflexivity in revisions is a positive step.
- However, the sample size justification weak, 10 participants is acceptable in qualitative research, but justification should reference e.g. saturation
- The interview guide is described but not appended as a supplement. Consider adding it as Supporting Information for reproducibility. Also, clarify if interviews were audio-recorded and how long they lasted.
- Sampling: Purposive approach is justified, but briefly note how variation was minimized for bias. Also, how recruitment was done and sampling criteria.
- In the analysis section Braun & Clarke’s six steps are described, but interpretation is limited. You could add information on coding process e.t.c.

**Results:**

- The section is well-supported with quotes and the themes are balanced. However, some themes overlap e.g. “perceived benefits” “personal health improvement”. Consider merging. Some quotes are also too long, consider making them concise.
- Ensure all quotes are anonymized consistently (e.g., avoid any identifiable details).

**Discussion and Conclusion**

- Effectively compares findings to literature. Implications for holistic care are strong.
- The author restates findings instead of interpreting them. You need to discuss your findings and show the so what…?
- To strengthen the paper, you can introduce a theoretical perspective
- Add a sentence on limitations (e.g., single-site study limits generalizability; potential researcher bias from nursing backgrounds, though reflexivity mitigates this). The conclusion repeats some abstract content; shorten for impact and emphasize calls to action.

**References:**

- Check for duplicates. Add page numbers for journal articles where missing.

**General Editorial Issues:**

- Language and Clarity: Overall clear, but proofread for minor grammatical issues and use scientific English. Ensure consistent terminology (e.g., CVDs vs. CVD - define on first use).
- Formatting: Ensure tables are inserted in-text (e.g., Insert Table 1 here on page 13).
- If applicable, upload the interview guide as a supplement.
