# Supplementary material for: Perceived facilitators and barriers influencing lifestyle modification among adults with cardiovascular disease attending a cardiac clinic at a referral hospital in northern Tanzania: An exploratory qualitative study
Source: PLOS Glob Public Health. 2026 Oct 27;6(10):e0007537. doi: 10.1371/journal.pgph.0007537 (PMC13712588; doi:10.1371/journal.pgph.0007537)
Supplement: Supplementary file 4 [file pgph.0007537.s007.docx]

The revised version seems considerably improved and is very close to being ready for acceptance. I don’t have any new methodological concerns, but minor copyediting is required before acceptance.

1. In the introduction on page 4, line 76, authors say that many studies “have primarily used qualitative approaches”. It should be quantitative approaches, as they are implying that these older studies measure and provide statistical clarity, while they are adding qualitative insight through this manuscript.
2. Page 6, lines 117 – 127 are repeated again in the paragraph below. Please remove one.
3. On page 14, line 308, the second theme, i.e., “**Barriers to lifestyle modification practices**” is labelled “Theme 4”. Instead, it should be Theme 2 as there are 2 final themes.
4. It seems like an editing note has been left in a participant quote “[what do you mean? Write it [e.g., correct information about lifestyle modification]”. Please correct this in line 361- 361. The same quote says “1 years” in line 361, which should be 1 year instead.
5. Som other issues that need correction:

- “CVDs-related deaths” in line 44, page 2, should be CVD-related deaths
- Please change “CVDs patients” on page 5, line 107 🡪 CVD patients
- Page 14, line 306: “cooker” should be cook instead
- Line 351, page 16, has a typo i.e. “meals.t”
- Line 412, page 19, “weight control” is repeated twice
