# Supplementary material for: Perceived facilitators and barriers influencing lifestyle modification among adults with cardiovascular disease attending a cardiac clinic at a referral hospital in northern Tanzania: An exploratory qualitative study
Source: PLOS Glob Public Health. 2026 Oct 27;6(10):e0007537. doi: 10.1371/journal.pgph.0007537 (PMC13712588; doi:10.1371/journal.pgph.0007537)
Supplement: Supplementary file 5 [file pgph.0007537.s008.docx]

**Response to editor and reviewers’ comments**

**Manuscript number: PGPH-D-25-02058R3**

**Revised title**: Perceived Facilitators and Barriers Influencing Lifestyle Modification among Adults with Cardiovascular Disease Attending a Cardiac Clinic at a Referral Hospital in Northern Tanzania: An Exploratory Qualitative Study

| **Section** | **Reviewer`s comment** | **Response** | **Page inserted** |
| --- | --- | --- | --- |
| **Reviewer #2** | | | |
| **Title and scope** | The title needs modification. The sample size is too small as the scope of the title is potentially broad. | Thank you for this important comment. We narrowed the title to accurately reflect the qualitative scope and focus on perceived influences on lifestyle modification. The revised title is: “Perceived Facilitators and Barriers Influencing Lifestyle Modification among Adults with Cardiovascular Disease Attending a Cardiac Clinic at a Referral Hospital in Northern Tanzania: A Qualitative Study.” We also clarified that the sample of 10 participants was guided by thematic saturation rather than statistical representativeness. | Page 1; Page 6 |
| **Materials and Methods** | The methods used are not clear and appropriate. | Thank you. We strengthened the Methods section by clarifying the exploratory qualitative design, eligibility criteria, purposive sampling, recruitment, thematic saturation, interview procedures, reflexivity, transcription/translation, coding, and thematic analysis. | Pages 5-10 |
| **Data analysis** | The reviewer indicated that the statistical analysis was not appropriate/rigorous. | We clarify that this was a qualitative study; therefore, inferential statistical analysis was not applicable. The manuscript now more clearly describes Braun and Clarke’s thematic analysis and the procedures used to enhance coding consistency and rigor. | Pages 8 |
| **Language and clarity** | The reviewer indicated that the manuscript was not presented in sufficiently standard English. | The manuscript was carefully proofread and edited for grammar, clarity, punctuation, and consistent scientific terminology throughout. | Throughout manuscript |
| **Reviewer #3** | | | |
| **Overall assessment** | All comments have been addressed. The manuscript is nicely written and the analysis of the qualitative results is effective. | We sincerely thank the reviewer for the positive assessment and for confirming that the previous comments were adequately addressed. | N/A |
| **Reviewer #4** | | | |
| **Introduction** | Lines 43–45: Please review the punctuation. Full stops appear to be missing after the references (2–4), LMICs (2,4), and 2030 (3,5). | Thank you. The missing punctuation was corrected in the Introduction. | Page 2 |
| **Study area** | Line 99: Please ensure consistency between the response letter and the revised manuscript. The term “faith-based” still appears in the Study area section, although the response indicated that it was removed. | Thank you for identifying this inconsistency. The term “faith-based” has now been removed from the Study Area section. | Page 6 |
| **Study population and eligibility criteria** | Line 110: A full stop is missing at the end of the sentence. | Corrected as suggested. | Page 6 |
| **Sample size and sampling procedure** | Lines 112–116: This paragraph appears redundant, as the same information is presented in greater detail immediately afterward. | We agree. The duplicated paragraph was removed and the sampling description was consolidated. | Page 6-7 |
| **Sample size and sampling procedure** | Lines 128–135: The explanation regarding data saturation, participant recruitment, and purposive sampling is largely repeated. Please consider consolidating these paragraphs. | Thank you. The repeated information on recruitment, saturation, and purposive sampling was consolidated into one concise paragraph. | Page 6-7 |
| **Sample size and sampling procedure** | Line 127: A full stop is missing at the end of the sentence. | Corrected as suggested. | Page 6-7 |
| **Data collection method and tool** | Lines 138–147: Some information overlaps with the subsequent Data collection procedure section. Please simplify this paragraph by focusing on the interview guide and avoiding repetition. | Thank you. This subsection was streamlined to focus on the semi-structured interview guide, while procedural details were retained under Data Collection Procedure. | Page 7 |
| **Data collection procedure** | Lines 166–169: The justification for the interview duration contains repeated wording. | We agree. The repeated wording was removed and the explanation of the 20–30 minute interview duration was made concise. | Page 7-8 |
| **Data analysis** | Line 177: Please rephrase the sentence to avoid repetition of the word root reflexivity/reflective. | The sentence was rephrased to improve readability while retaining the explanation of researcher positionality and reflexive analysis. | Page 8 |
| **Results** | Lines 228–232: Please review the punctuation separating the two main themes. A full stop appears to be missing after “…guidance from healthcare providers”. | Corrected. The two main themes are now clearly separated and appropriately punctuated. | Page 10 |
| **Results** | Several quotations are presented consecutively without transition sentences or clear separation. Please add brief transition sentences and review punctuation/formatting. | Thank you. Brief analytical and transition sentences were added after key quotations to improve narrative flow and show how quotations illustrate the broader themes. | Pages 10–17 |
| **Terminology** | Please perform a final consistency check. Expressions such as “CVDs patients” are still present. | A final terminology check was completed. Inappropriate expressions such as “CVDs patients” were replaced with “patients with CVD,” “CVD patients,” or “adults with CVD,” as appropriate. | Throughout manuscript |
| **Discussion** | Line 412: Please remove the duplicated expression “weight control” and revise the repeated use of “outcomes” within the same sentence. | Corrected. The duplicated and unsupported reference to weight control was removed, and the sentence now refers only to outcomes supported by the Results, including improved energy, reduced breathlessness, lower blood glucose, and normalized blood pressure. | Page 18 |
| **Reviewer #5** | | | |
| **Introduction** | Page 4, line 76: “have primarily used qualitative approaches” should be “quantitative approaches.” | We agree. “Qualitative approaches” was corrected to “quantitative approaches.” | Page 4 |
| **Sample size and sampling procedure** | Page 6, lines 117–127 are repeated again in the paragraph below. Please remove one. | Thank you. The duplicated text was removed and the sampling and saturation information was consolidated. | Page 6-7 |
| **Results** | Page 14, line 308: “Barriers to lifestyle modification practices” is labelled “Theme 4.” It should be Theme 2. | Corrected. The barriers theme is now consistently labelled “Theme 2” in the Results and Table 2. | Page 10; Page 29 |
| **Results - participant quotation** | Line 361: An editing note “[what do you mean? …]” has been left in a participant quote. The same quote says “1 years,” which should be “1 year.” | Thank you for identifying this error. The internal editing note was removed and the participant duration was corrected to “1 year.” The quotation now reads: “No, I have never received any [information about lifestyle modification].” | Page 16 |
| **Introduction – terminology** | “CVDs-related deaths” should be “CVD-related deaths.” | Corrected as suggested. | Page 2 |
| **Terminology** | Please change “CVDs patients” to “CVD patients.” | Corrected throughout the manuscript using consistent terminology such as “patients with CVD” or “adults with CVD.” | Throughout manuscript |
| **Results – quotation** | Page 14, line 306: “cooker” should be “cook.” | Corrected as suggested. | Page 13 |
| **Results – quotation** | Line 351: There is a typo “meals.t”. | Corrected to “meals.” | Page 16 |
| **Discussion** | Line 412: “weight control” is repeated twice. | Corrected. In addition, because weight control was not reported in the Results, the unsupported claim was removed from the Discussion. | Page 19 |
| **Reviewer #6** | | | |
| **Overall/Discussion** | The Discussion lacks sufficient critical analysis and interpretation and reads more as a descriptive report than a qualitative study offering deeper conceptual insights. | Thank you for this important comment. The Discussion was substantially revised to explain why the findings may occur, how personal, household, clinical, social, and economic influences interact, and what the study contributes beyond previous descriptive evidence. | Pages 18–23 |
| **Introduction** | Lines 74–77: Did you mean to say quantitative rather than qualitative approaches? | Yes. This was an error and has been corrected to “quantitative approaches.” | Page 5 |
| **Introduction** | Lines 81–83: Knowledge is only one determinant of behavior; acknowledge self-efficacy, motivation, social norms, environmental constraints, access to resources, and health-system influences. | We agree. The Introduction now explicitly explains that knowledge is only one determinant of behavior and that the knowledge–practice gap reflects interacting personal, social, environmental, resource, and health-system influences. | Page 4-5 |
| **Materials and Methods** | Lines 128–136 and 150–152: These statements are repeated earlier verbatim. | Thank you. Repeated information on recruitment, saturation, sampling, and data collection was removed and consolidated. | Pages 5–8 |
| **Results** | Some themes can be grouped together, like religious and social support. | We agree. Overlapping facilitator sub-themes were consolidated. Social, family, and spiritual support are now presented together, while healthcare guidance remains analytically distinct. | Pages 10–17; Page 29 |
| **Results** | Results reiterate participants’ quotes followed by author rewriting. Add one or two interpretive sentences after quotations to explain how the quotation illustrates the broader theme. | Thank you. Concise interpretive statements were added after key quotations to explain their analytical meaning rather than simply restating participant words. | Pages 10–17 |
| **Discussion** | The Discussion needs greater insight into why the findings occurred, how the Tanzanian context influences them, and what the study contributes beyond previous literature. | We substantially strengthened the Discussion by interpreting shared household food practices, affordability, physical limitations, psychological stress, family support, variable counselling, and community influences within the Tanzanian referral-care context. | Pages 18–23 |
| **Practical implications** | Practical implications are too general/generic. | Practical implications were made more specific, including repeated behavior-specific counselling, symptom-adapted activity planning, psychosocial assessment, family-inclusive counselling, assessment of food affordability, and theory-informed intervention development. | Page 18-23 |
| **Limitations** | Missing limitations include single referral hospital, potential selection bias, social desirability bias, possible recall bias, cross-sectional interviews, and limited transferability. | We agree. The Limitations section now explicitly addresses all these issues, as well as the broad interview-guide wording, translation-related loss of nuance, and researcher positionality. | Page 21-22 |
| **Conclusion** | The Conclusion should include implications for clinical practice, future intervention development, and future qualitative or mixed-methods research. | The Conclusion was revised accordingly and now includes specific implications for clinical practice, theory-informed intervention development, and future multi-site qualitative, longitudinal, and mixed-methods research. | Page 22 |
| **Reviewer #7** | | | |
| **Results - participant characteristics** | The text states that duration since diagnosis ranged from six months to fifteen years, but the maximum in Table 1 is 14 years. | Thank you. The Results were corrected to state that duration since CVD diagnosis ranged from six months to 14 years, consistent with Table 1. | Page 10; Pages 28 |
| **Introduction** | “A high burden of NCDs, with approximately 33%…” is unclear. Specify 33% of what. | We agree. The sentence was revised to specify that NCDs account for approximately 33% of all deaths in Tanzania, while CVDs account for approximately 13% of all deaths. | Page 3 |
| **Results - Lack of guidance** | The P3 quotation describes receiving explanations from doctors at the clinic, which contradicts the sub-theme. Either move it or explain that experiences of guidance were mixed. | Thank you. The P3 quotation was removed from the “Lack of guidance” sub-theme. The narrative now clarifies that experiences were mixed and that the barrier concerned inconsistency in the depth, continuity, and follow-up of lifestyle counselling. | Page 16 |
| **Results - participant quotation** | The P1 quotation contains an unresolved internal query between co-authors. | We apologize for the oversight. The internal editing query was removed, and the quotation was corrected to: “No, I have never received any [information about lifestyle modification].” | Page 16 |
| **Results - thematic structure** | Overlapping sub-themes remain: “Personal health improvement” has been moved under “Perceived benefits and outcomes,” and “Motivation for lifestyle changes” duplicates “Medical advice and counselling.” | We agree. Repetitive material under perceived benefits was reduced, and overlapping facilitator sub-themes were consolidated. Healthcare-related motivation is now incorporated within medical advice and counselling rather than presented as a separate overlapping sub-theme. | Pages 10–13; Page 29 |
| **Discussion** | The Discussion mentions “weight control,” although this was not reported in the Results. Please evidence or remove it and correct the duplication. | Thank you. The unsupported reference to weight control was removed. The Discussion now refers only to outcomes supported by participant accounts, such as improved energy, reduced breathlessness, lower blood glucose, and normalized blood pressure. | Page 18-22 |
| **Reviewer #8** | | | |
| **Title and study focus** | The interview guide appears more relevant to “Influences on lifestyle modifications” than to identifying lifestyle modification “practices.” This is also relevant to the title. | We agree. The manuscript was reframed to focus on perceived facilitators and barriers influencing lifestyle modification rather than claiming to systematically assess lifestyle modification practices. The title was revised accordingly. | Page 1; Page 7 |
| **Data collection instrument** | The broad term “lifestyle modifications” may have been understood differently by interviewees. Some questions appear leading, and two are yes/no questions without evident follow-up inquiry. | Thank you. Because data collection was already completed, the guide could not be retrospectively redesigned. We therefore narrowed the claims of the manuscript | Page 7 |
| **Introduction and Discussion** | Diet, physical activity, smoking, and alcohol consumption should be discussed more fully because facilitators and barriers vary according to the specific behavior. | We agree. The Introduction and Discussion now explicitly identify these key behaviors and explain that determinants differ by behavior—for example, diet may be constrained by household cooking and food affordability, whereas physical activity may be limited by symptoms and functional capacity. | Pages 3-4; Pages 18-22 |
| **Behavior change theory** | The relevance of Social Cognitive Theory should be discussed further, particularly because it was added after review rather than used prospectively in the study design. | Thank you. We now explicitly state that Social Cognitive Theory was not used to design the interview guide but is applied cautiously as a post hoc interpretive lens. The Discussion relates outcome expectations, self-efficacy, and social reinforcement to participants’ accounts. | Page 18 |
| **Behavior change theory/COM-B** | Consider the COM-B model and Behavior Change Wheel to analyze influences and guide future intervention development. | We appreciate this suggestion. The Discussion now maps the findings to capability, opportunity, and motivation and explains how COM-B and the Behavior Change Wheel can guide behavior-specific intervention functions. The recommended Michie et al. reference was added. | Page 19; Page 27 |
| **Prevention terminology** | Differentiate primary, secondary, and tertiary prevention behaviors. Lifestyle behaviors among people with established CVD are tertiary prevention in the classical public-health framework. | Thank you. The Introduction now clarifies that participants already had established CVD and that the behaviors explored are aimed at reducing progression, complications, and recurrent events. This is described as tertiary prevention in the classical framework while acknowledging secondary-prevention terminology commonly used in cardiovascular care. | Page 3 |
| **Introduction** | Line 53: “NCDs account for 33%” is unclear. Of what? | Corrected. The sentence now states that NCDs account for approximately 33% of all deaths in Tanzania. | Page 3 |
| **Introduction – grammar** | Line 87: “Patients’” – add apostrophe. | Corrected as suggested. | Page 4 |
| **Sampling - grammar** | Line 129: Clinic. | Corrected as suggested. | Page 6 |
| **Eligibility criteria** | Lines 129–131: Inclusion criteria should be moved to the “Study population and eligibility criteria” section. | We agree. The eligibility criteria are now presented only in the appropriate Study Population and Eligibility Criteria subsection, and duplication in the sampling section was removed. | Page 6 |
| **Future research and intervention development** | Future research should be based on recognizable behavioral frameworks and well-designed, pretested instruments. | We appreciate this recommendation. The Discussion and Conclusion now emphasize theory-informed, behavior-specific future research using appropriately developed and pretested qualitative instruments, with Social Cognitive Theory and COM-B identified as useful frameworks. | Pages 20–22 |
