# Supplementary material for: Perceived facilitators and barriers influencing lifestyle modification among adults with cardiovascular disease attending a cardiac clinic at a referral hospital in northern Tanzania: An exploratory qualitative study
Source: PLOS Glob Public Health. 2026 Oct 27;6(10):e0007537. doi: 10.1371/journal.pgph.0007537 (PMC13712588; doi:10.1371/journal.pgph.0007537)
Supplement: Supplementary file 6 [file pgph.0007537.s009.docx]

**Response to editor and reviewers’ comments**

**Manuscript number: PGPH-D-25-02058R3**

**Title**: Perceived Facilitators and Barriers Influencing Lifestyle Modification among Adults with Cardiovascular Disease Attending a Cardiac Clinic at a Referral Hospital in Northern Tanzania: An Exploratory Qualitative Study

| **Section** | **Comment** | **Response** | **Page inserted** |
| --- | --- | --- | --- |
| **Editor and Journal requirements** | | | |
| **References** | Please review the reference list to ensure that it is complete and correct. Remove or justify retracted articles where applicable, and mention reference changes in the rebuttal. | All the references were checked for accuracy and completeness. Specifically, the DOI for reference numbers 19 and 21 was rechecked and edited for accuracy | Page 23-27 |
| **Reviewer #3** | | | |
| **Overall assessment** | All comments have been addressed. The ethical issues are clearly stated, the methodology is described in detail, and the manuscript can be published. | We thank the reviewer for the positive assessment and for confirming that the previous comments were addressed. The descriptions of methodology and ethical safeguards have been retained. | Methods, lines 109–207 |
| **Reviewer #4** | | | |
| **Overall assessment** | The main substantive and methodological concerns have been satisfactorily addressed, and the manuscript has improved considerably. After the remaining minor corrections, the manuscript will be suitable for publication. | Thank you for your positive assessment of the substantive and methodological revisions and for noting the improvement in the manuscript. We appreciate your careful review. | N/A |
| **Introduction** | Line 45: “strokes (2-4) Alarmingly”; line 46: “countries (LMICs) (2,4) If effective”; lines 46–47: “projected to increase to 23 million by 2030 (3,5) Tanzania is”. Full stops remain missing after the citations. | Thank you for identifying these persistent errors. Full stops are now present after “(2-4)”, “(2,4)”, and “(3,5)”. We checked each correction directly in the revised manuscript. | Page 2, lines 45–46; page 3, line 47 |
| **Study area** | Line 119: “KCMC, a faith-based, zonal referral and teaching hospital...”. The term “faith-based” remains despite the earlier response stating that it was removed. | The term “faith-based” has been removed. The sentence now describes KCMC as “a zonal referral hospital in northern Tanzania located in Kilimanjaro region.” | Page 6, lines 117–118 |
| **Interview guide** | Line 140: “developed to explore participants’ perceived influences on lifestyle modification among.” The word “among” is extraneous and the sentence is incomplete. | We corrected the incomplete sentence to read: “Data were collected using a semi-structured interview guide developed to explore participants’ perceived influences on lifestyle modification among adults with CVD.” | Page 7, lines 138–139 |
| **Participant quotation** | “meals.t” should be corrected to “meals.” | Corrected. The quotation now ends with “during meals.” | Page 16, lines 339–340 |
| **Participant descriptor** | “1 years with CVD” should be corrected to “1 year with CVD.” | Corrected. The descriptor now reads “P1, 1 year with CVD”. | Page 16, lines 352–353 |
| **Response consistency** | Carefully cross-check the final manuscript against the response letter because corrections reported as completed still remain in the manuscript. | We cross-checked the specific corrections against the final manuscript. The missing full stops, “faith-based”, incomplete “among” sentence, “meals.t”, and “1 years with CVD” have all been corrected. The Data collection method and tool subsection uses the singular “interview guide”. | Lines 45–47, 117–118, 138–139, 339–340 and 352–353 |
| **Reviewer #5** | | | |
| Participant descriptor | Page 16, line 352: “P1, 1 years with CVD” should be “P1, 1 year with CVD.” | Corrected as suggested. | Page 16, lines 352–353 |
| Participant quotation | Page 15, line 340: “during meals.t.” should be “during meals.” | Corrected as suggested. | Page 16, lines 339–340 |
| Abstract objective | “among adults with cardiovascular patients attending the cardiac clinic” is incorrect. Please revise to “among adults with cardiovascular disease attending the cardiac clinic” or similar wording. | The Abstract objective now refers to “adults with cardiovascular disease attending the cardiac clinic at Kilimanjaro Christian Medical Centre (KCMC)”. | Page 1, lines 19–21 |
| Data availability | In response to the data-availability question, the reviewer selected “No”. | We clarify that a Data Availability Statement is provided in the online submission system. It explains the confidentiality restrictions on public release of full interview datasets and identifies a non-author institutional contact for requests for de-identified data: the Directorate of Research and Publications, MUHAS (drp@muhas.ac.tz). The complete statement is reproduced below. The supporting data described in that statement are provided in the manuscript and Supporting Information files. | Online submission system and Supporting Information; no manuscript line number |
| **Reviewer #8** | | | |
| **Interview guide wording** | Line 140: the word “among” is dangling; please review the English. | The sentence has been completed by specifying “among adults with CVD”, and the wording was checked for grammatical clarity. | Page 7, lines 138–139 |
| **Guide or guides** | Line 140: guides or guide? | The Data collection method and tool subsection now uses the singular “semi-structured interview guide” and subsequently refers to “the guide”, reflecting the single interview instrument used. | Page 7, lines 138–140 |
| **Exercise costs** | Line 376: Gym membership should not be included as a valid financial constraint. Walking and floor exercises at home without equipment are useful. If gym membership is retained, explain that it is not a valid constraint to exercise. | We agree that gym membership is not required for physical activity. Gym membership has been removed from the authors’ list of lifestyle-modification costs. The participant’s quotation is retained as an account of a perceived barrier, rather than evidence that exercise necessarily requires payment. | Page 17, lines 376–383; page 18, lines 384–386 |
| **Knowledge and behavior change** | Line 412: “partly aligns”: authors should be more adamant that information and knowledge do not themselves lead to behavior change without other interventions. | We removed “partly aligns” and explicitly state that information and knowledge alone are insufficient to produce or sustain lifestyle behavior change. The Discussion explains that translating knowledge into practice requires additional support addressing motivation, physical capability, social and family influences, financial constraints, and environmental opportunities. This interpretation is linked to COM-B, and reference 29 is retained briefly to contextualize supportive interventions. | Page 19, lines 407–423 |
| **Data availability** | In response to the data-availability question, the reviewer selected “No”. | We clarify that a Data Availability Statement is provided in the online submission system. It explains the confidentiality restrictions on public release of full interview datasets and identifies a non-author institutional contact for requests for de-identified data: the Directorate of Research and Publications, MUHAS (drp@muhas.ac.tz). The complete statement is reproduced below. The supporting data described in that statement are provided in the manuscript and Supporting Information files. | Online submission system and Supporting Information |
