# Supplementary material for: Perceived facilitators and barriers influencing lifestyle modification among adults with cardiovascular disease attending a cardiac clinic at a referral hospital in northern Tanzania: An exploratory qualitative study
Source: PLOS Glob Public Health. 2026 Oct 27;6(10):e0007537. doi: 10.1371/journal.pgph.0007537 (PMC13712588; doi:10.1371/journal.pgph.0007537)
Supplement: Supplementary file 7 [file pgph.0007537.s004.doc]

**Response to editor and reviewers’ comments**

| **Section** | **Reviewer`s comment** | **Response** | **Page inserted** |
| --- | --- | --- | --- |
| **Journal requirements** | | | |
| 1. | We have amended your Competing Interest statement to comply with journal style. We kindly ask that you double check the statement and let us know if anything is incorrect. | The competing interest statement has been double-checked and revised to comply with journal's style | Pg 23 |
| 2. | Please provide a/amend your detailed Financial Disclosure statement. This is published with the article. It must therefore be completed in full sentences and contain the exact wording you wish to be published. | The financial statement disclosure statement has been amended and completed in full | Pg 23 |
| 3. | The data that support the findings of this study are not openly available due to reasons of sensitivity and available from the corresponding author upon reasonable request | I understand the importance of data sharing but due to unavoidable ethical reasons, the **data sets for this study are available upon request.** |  |
| 4. | If the reviewer comments include a recommendation to cite specific previously published works, please review and evaluate these publications to determine whether they are relevant and should be cited. There is no requirement to cite these works unless the editor has indicated otherwise. | Noted with thanks  There is no recommendation to cite specific previously published works |  |
| **Reviewer #1** | | | |
| **Introduction** | Provides global/Tanzanian context with citations; builds rationale for study gap. Logical flow from CVD burden to lifestyle modification importance. | The background section has been improved with further description of both global and Tanzanian context with additional of more citations. | 2,3,4 |
|  | Could be concise; some repetition (e.g., CVD stats). Gap statement: Claims "paucity of research" in Tanzania, support with a brief literature search summary. | Thank you for the comment.  This has been addressed with clarity, removal of repetitions, and addition of a brief literature search showing paucity of research in Tanzania | Pg 3,4 |
|  | Grammar- "modest alcoholism" - "moderate alcohol consumption"; "significantly rise" - "significant rise". | Thank you for the observation.  This has been addressed by ensuring clarity and consistency, and the use of correct grammars | Pg 3 line 52 |
|  | Strengthen with 1-2 sentences on qualitative approaches' value in LMICs. | Thank you for the suggestion.  There are limited qualitative studies on lifestyle modification among CVD patients conducted in LMICs, however, we managed to get one and we have included it in the introduction section | Pg 3-4, lines 64-68 |
| **Materials and Methods** | Sampling: n=10 is small but justified by saturation; however, describe recruitment variation (e.g., how ensured diversity in CVD types, age, gender? 60% female noted in results-move here?). | Thank you for the comment.  We have clarified that participants were purposively selected to ensure diversity in gender, duration of illness, and cardiovascular conditions to capture a wide range of perspectives. Ooh so sorry, 60% is not for this part but for results hence deleted from here. | Pg 4,5 line 104-112 |
|  | Data collection: Tool (interview guide) mentioned but not appended or described (e.g., sample questions). Interviews 20-30 min is a bit short for in-depth; justify. No mention of pilot testing or reflexivity (e.g., researcher bias as nurses/clinicians?). | Thank you for the comment.  The data collection tool was further described to include sample questions. More justification was given for the time of 20-30 minutes used in the in-depth study. We have indicated that the interview guide was piloted with two participants to ensure clarity.  Reflexivity has been acknowledged accordingly in the methodology within data collection and analysis sections to address potential researcher influence on data collection, analysis and interpretation. | Pg 5,6 |
|  | Analysis: Steps outlined, but lacks detail on software (if used, e.g., NVivo), inter-rater reliability (e.g., how supervisors refined themes), or member checking for validity. | Thank you for the observation.  Further explanation was given that the analysis was done manually without the use of any software. | Pg 7 |
|  | Study period: May-June 2024 in abstract; May-July in text take note of the inconsistency. | Thank you for the comment.  We have addressed this accordingly | Pg 1,6 |
| **Results** | Good | Thank you for the compliment | 8-16 |
| **Discussions** | Good; Links findings to literature; highlights implications (e.g., tailored education). Contrasts with global studies effectively. | Thank you for very much | 18-21 |
|  | Structure: Starts with summary but jumps between facilitators/barriers; maybe organize by theme? | Thank you for the comment. To avoid confusion, this has been organized by theme | 18-21 |
|  | Synthesis: Comparisons (e.g., to refs 24-28) are surface level; deepen (e.g., how Tanzanian cultural factors differ from Ethiopian study in ref 4?) | Thank you, this part has been deepen as advised | 18-21 |
|  | Novelty: Claims alignment but doesn't emphasize unique contributions (e.g., religious support in faith-based setting). | Thank you for the clarification |  |
| **Conclusion** | Good; Reiterates key findings and calls for action (e.g., personalized education), | Thank you very much | 22 |
|  | Repetitive of abstract/discussion; too long for a concise wrap-up. Need to add implications for practice/policy. | We have shortened and refined the conclusion to summarize the study succinctly and added explicit implications for practice and policy | Pg 22 |
|  | Language and Style: A couple of errors (e.g., "life modification" - "lifestyle"; "i couldn’t" - "I couldn't"; run-on sentences). Proofread thoroughly or use editing tools. Enhance methodological details (rigor, reflexivity); fix inconsistencies (dates); deepen discussion synthesis. | - The manuscript has been thoroughly proofread and edited for grammatical accuracy, clarity, and coherence.  -Thanks for the advice of using editing tool.  -Both rigor and reflexivity have been addressed in previous pages within methodology section.  -All dates made consistent.  -Discussion synthesis has been deepened | Pg 1-27 |
|  | PLOS authors have the option to publish the peer review history of their article ([what does this mean?](https://track.editorialmanager.com/CL0/https:%2F%2Fjournals.plos.org%2Fglobalpublichealth%2Fs%2Feditorial-and-peer-review-process%23loc-peer-review-history/1/010f0199707f1783-de577588-9771-49b1-8a75-fe8cef564000-000000/xTwVq1MLOI03IaFyTS1UeNwQPxy1KCDs4GfuSYrw-tk=229)). If published, this will include your full peer review and any attached files. | Noted with thanks |  |
|  | **Do you want your identity to be public for this peer review?** If you choose “no”, your identity will remain anonymous but your review may still be made public. | Noted with thanks |  |
|  | | | |
