# Supplementary material for: Perceived facilitators and barriers influencing lifestyle modification among adults with cardiovascular disease attending a cardiac clinic at a referral hospital in northern Tanzania: An exploratory qualitative study
Source: PLOS Glob Public Health. 2026 Oct 27;6(10):e0007537. doi: 10.1371/journal.pgph.0007537 (PMC13712588; doi:10.1371/journal.pgph.0007537)
Supplement: Supplementary file 8 [file pgph.0007537.s006.doc]

**Response to editor and reviewers’ comments**

| **Section** | **Reviewer`s comment** | **Response** | **Page inserted** |
| --- | --- | --- | --- |
| **Reviewer #1** | | | |
| **Title** | The title is too long consider removing faith-based unless it is central to analysis | The title was refined by removing the word “faith-based.” | Page 1 |
| **Abstract and Keywords** | - There grammar issues e.g. where you state “there are limited research exploring lifestyle modification practices…” - The abstract is concise but could more explicitly state the sample size (n=10) and key sub-themes for facilitators and barriers. The conclusion is vague. Consider revising to show clearer implications of study. - Keywords are appropriate (cardiovascular disease; Lifestyle Modification; Behavioral Change") but add "Tanzania" or "qualitative study" for better indexing. | - Grammatical issues were corrected - Sample size was explicitly stated, key sub-themes for facilitators and barriers were stated in the results sub-section of the abstract - Conclusion was refined and revised to show more clearly the implications of the study - “Tanzania” and “Qualitative study” was added as part of key words | Page 1-2 |
| **Introduction** | A strong global and local context has been highlighted (e.g., 13% of NCD deaths in Tanzania attributed to CVDs). However, fix minor formatting inconsistencies in citations, e.g., “(2).(3,4)” on page 5 should be “(2–4)” for consistency. Several paragraphs repeat the same ideas e.g when you talk about CVD burden. | Formatting for citations was done accordingly for the whole document e.g. “2-4” instead of “(2).(3,4)” | Page 2-4 |
|  | There are grammar issues for instance when you say… “Studies conducted in African countries show that the adherence to a healthier lifestyle for patients at risk of CVDs ranges between 16.4%-52.7%.” Consider rephrasing. | Statement was rephrased as: “ Studies across African countries have demonstrated suboptimal adherence to healthy lifestyle practices among patients at risk of CVDs, with reported adherence levels ranging from 16.4% to 52.7%” | Page 4 |
|  | The gap analysis is unclear (e.g., quantitative studies exist, but qualitative narratives are lacking). Consider highlighting what other studies have/whet they missed and why the qualitative evidence is needed. | The study gap was revised by highlighting what other studies have/what they missed, and why there is a need for qualitative evidence | Page 4-5 |
| **Materials and Methods** | - The section is detailed and transparent. The addition of reflexivity in revisions is a positive step. - However, the sample size justification weak, 10 participants is acceptable in qualitative research, but justification should reference e.g. saturation | Thank you for this important comment. We have strengthened the sample size justification in the Methods section by clarifying that the final sample size was determined based on data saturation, which is an established principle in qualitative research. We further explained that interviews continued until no new themes or substantive insights emerged, with saturation achieved after eight interviews and two additional interviews conducted to confirm thematic consistency and adequacy | Page 6 |
|  | The interview guide is described but not appended as a supplement. Consider adding it as Supporting Information for reproducibility. Also, clarify if interviews were audio-recorded and how long they lasted. | Thank you for this valuable comment. We have revised the manuscript to clarify that all interviews were audio-recorded and lasted approximately 20–30 minutes. We further explained that, although relatively brief, the interview duration was sufficient to generate rich and meaningful data through the use of semi-structured and probing questions. In addition, the interview guide has now been included as Supporting Information to enhance transparency and reproducibility of the study | Page 7-9 |
|  | Sampling: Purposive approach is justified, but briefly note how variation was minimized for bias. Also, how recruitment was done and sampling criteria. | Thank you for this insightful comment. We have revised the sampling section to provide additional details on the recruitment process, inclusion criteria, and purposive sampling strategy. Specifically, we clarified that participants were recruited from the outpatient cardiac clinic based on predefined eligibility criteria, including age, duration since CVD diagnosis, ability to communicate in Swahili, and willingness to participate. We also explained that variation was enhanced by purposively recruiting participants across different age groups, gender, duration of illness, and types of cardiovascular diseases to minimize selection bias and capture diverse experiences. Furthermore, we strengthened the justification for the sample size by elaborating on the process of achieving thematic saturation. | Page 6-7 |
|  | In the analysis section Braun & Clarke’s six steps are described, but interpretation is limited. You could add information on coding process e.t.c. | Thank you for this valuable comment. We have revised the data analysis section to enhance clarity on the coding and interpretation process. Specifically, we have added details on how the coding was conducted independently by GNM and BM, how discrepancies were resolved through discussion to improve consistency, and how themes were collaboratively developed and refined by the research team. We also clarified the roles of each author during transcription, translation, coding, and theme development, and highlighted the involvement of experienced qualitative researchers in reviewing and refining the themes to strengthen rigor, credibility, and confirmability of the analysis | Page 9-10 |
| **Results** | - The section is well-supported with quotes and the themes are balanced. However, some themes overlap e.g. “perceived benefits” “personal health improvement”. Consider merging. Some quotes are also too long, consider making them concise. - Ensure all quotes are anonymized consistently (e.g., avoid any identifiable details). | Thank you for these valuable comments. We revised the Results section by merging overlapping sub-themes to improve thematic clarity and reduce redundancy. Specifically, overlapping sub-themes such as ‘perceived benefits’ and ‘personal health improvement’ were reviewed and merged to avoid redundancy and enhance thematic distinction. We also shortened lengthy quotations for conciseness while retaining their original meaning. Furthermore, all quotations were reviewed to ensure consistent anonymization and removal of potentially identifiable details | Page 14-20 |
| **Discussion and Conclusion** | Effectively compares findings to literature. Implications for holistic care are strong. | Thank you for compliment | Page 21-25 |
|  | The author restates findings instead of interpreting them. You need to discuss your findings and show the so what…? | We have revised the Discussion section to provide deeper interpretation of the findings beyond restating the results by emphasizing their practical and contextual implications for cardiovascular care in Tanzania and similar resource-limited settings. We also strengthened the ‘so what’ aspect by explaining how the findings inform patient-centered, psychosocial, and community-based interventions | Page 21-25 |
|  | To strengthen the paper, you can introduce a theoretical perspective | We have introduced the Social Cognitive Theory to provide a theoretical perspective for understanding the interaction between personal beliefs, social support, and lifestyle modification behaviors among cardiovascular patients | Page 22 |
|  | Add a sentence on limitations (e.g., single-site study limits generalizability; potential researcher bias from nursing backgrounds, though reflexivity mitigates this). The conclusion repeats some abstract content; shorten for impact and emphasize calls to action. | Thank you for this important comment. We have revised the limitations section by explicitly acknowledging that the single-site study design may limit the transferability of the findings to other settings. We also clarified the potential influence of researcher bias due to the researchers’ nursing and clinical backgrounds, while emphasizing that reflexivity measures, including reflective discussions and field notes, were used to minimize this influence during data collection and analysis  We have revised the conclusion to make it more concise and action-oriented by removing repetition and strengthening the key implications for practice and policy, with clearer emphasis on patient-centered, culturally sensitive, and resource-supported cardiovascular care interventions | Page 26 |
| **References** | Check for duplicates. Add page numbers for journal articles where missing. | Thank you for this comment. We have carefully reviewed the reference list to identify and remove any duplicate entries. In addition, we have updated all journal article references to include missing page numbers where applicable to ensure completeness and adherence to the journal referencing guidelines | Page 28-32 |
| **General editorial issues** | Language and Clarity: Overall clear, but proofread for minor grammatical issues and use scientific English. Ensure consistent terminology (e.g., CVDs vs. CVD - define on first use). | Thank you for this helpful comment. We have thoroughly proofread the manuscript to correct minor grammatical errors and improve clarity using appropriate scientific English. We have also ensured consistent use of terminology throughout the manuscript, including defining cardiovascular disease (CVD) at first use and maintaining consistent usage of the term thereafter | N/A |
|  | Formatting: Ensure tables are inserted in-text (e.g., Insert Table 1 here on page 13). | Thank you for this comment. We have revised the manuscript to comply with PLOS ONE formatting requirements by inserting table placement indicators (e.g., ‘Insert Table 1 here’) within the main text at appropriate points. In addition, all tables have been moved to appear after the References section as required by the journal guidelines | Page 11-12 |
|  | If applicable, upload the interview guide as a supplement. | Thank you for this comment. We have now uploaded the semi-structured interview guide as a supplementary file to enhance transparency and support reproducibility of the study methods | NA |
|  | | | |
