# Supplementary material for: Perceived facilitators and barriers influencing lifestyle modification among adults with cardiovascular disease attending a cardiac clinic at a referral hospital in northern Tanzania: An exploratory qualitative study
Source: PLOS Glob Public Health. 2026 Oct 27;6(10):e0007537. doi: 10.1371/journal.pgph.0007537 (PMC13712588; doi:10.1371/journal.pgph.0007537)
Supplement: Peer review history

# Peer review: Decision Letter 0

**DOI:** 10.1371/journal.pgph.0007537.r001

**Authors:** Katherine Demi Kokkinias

22 Sep 2025

PGPH-D-25-02058

Facilitators and Barriers to Lifestyle Modification Practices among Cardiovascular Patients attending Cardiac Clinic at a Faith Based Hospital in Northern Tanzania

PLOS Global Public Health

Dear Dr. Mgala,

Thank you for submitting your manuscript to PLOS Global Public Health. After careful consideration, we feel that it has merit but does not fully meet PLOS Global Public Health’s publication criteria as it currently stands. Therefore, we invite you to submit a revised version of the manuscript that addresses the points raised during the review process.

==============================

Please note that we have only been able to secure a single reviewer to assess your manuscript. We are issuing a decision on your manuscript at this point to prevent further delays in the evaluation of your manuscript. Please be aware that the editor who handles your revised manuscript might find it necessary to invite additional reviewers to assess this work once the revised manuscript is submitted. However, we will aim to proceed on the basis of this single review if possible.

The reviewer raised a number of concerns that need attention.  Specifically, there were several concerns with the reporting of the methodology. Additionally, the reviewer had concerns with the grammar and language of the manuscript. The reviewer suggested ways to improve the flow of the article and improve the discussion.

Could you please revise the manuscript to carefully address the concerns raised?

==============================

Please submit your revised manuscript by Nov 03 2025 11:59PM. If you will need more time than this to complete your revisions, please reply to this message or contact the journal office at globalpubhealth@plos.org. When you're ready to submit your revision, log on to https://www.editorialmanager.com/pgph/ and select the 'Submissions Needing Revision' folder to locate your manuscript file.

Please include the following items when submitting your revised manuscript:

- A rebuttal letter that responds to each point raised by the editor and reviewer(s). You should upload this letter as a separate file labeled 'Response to Reviewers'.
- A marked-up copy of your manuscript that highlights changes made to the original version. You should upload this as a separate file labeled 'Revised Manuscript with Track Changes'.
- An unmarked version of your revised paper without tracked changes. You should upload this as a separate file labeled 'Manuscript'.

Guidelines for resubmitting your figure files are available below the reviewer comments at the end of this letter.

We look forward to receiving your revised manuscript.

Kind regards,

Katherine Demi Kokkinias, Ph.D.

Staff Editor

PLOS Global Public Health

Journal Requirements:

1. We have amended your Competing Interest statement to comply with journal style. We kindly ask that you double check the statement and let us know if anything is incorrect.

2. Please provide a/amend your detailed Financial Disclosure statement. This is published with the article. It must therefore be completed in full sentences and contain the exact wording you wish to be published.

**Please only choose the relevant sentences from below**

a. Please clarify all sources of funding (financial or material support) for your study. List the grants (with grant number) or organizations (with url) that supported your study, including funding received from your institution.

b. State the initials, alongside each funding source, of each author to receive each grant.

c. State what role the funders took in the study. If the funders had no role in your study, please state: “The funders had no role in study design, data collection and analysis, decision to publish, or preparation of the manuscript.”

d. If any authors received a salary from any of your funders, please state which authors and which funders.

If you did not receive any funding for this study, please simply state: “The authors received no specific funding for this work.

3.**(** The data that support the findings of this study are not openly available due to reasons of sensitivity and available from the corresponding author upon reasonable request)

**We note that you have indicated that there are restrictions to data sharing for this study. For studies involving human research participant data or other sensitive data, we encourage authors to share de-identified or anonymized data. However, when data cannot be publicly shared for ethical reasons, we allow authors to make their data sets available upon request. For information on unacceptable data access restrictions, please see http://journals.plos.org/plosone/s/data-availability#loc-unacceptable-data-access-restrictions.**

Before we proceed with your manuscript, please address the following prompts:

a) If there are ethical or legal restrictions on sharing a de-identified data set, please explain them in detail (e.g., data contain potentially identifying or sensitive patient information, data are owned by a third-party organization, etc.) and who has imposed them (e.g., a Research Ethics Committee or Institutional Review Board, etc.). Please also provide contact information for a data access committee, ethics committee, or other institutional body to which data requests may be sent.

b) If there are no restrictions, please upload the minimal anonymized data set necessary to replicate your study findings to a stable, public repository and provide us with the relevant URLs, DOIs, or accession numbers. Please see http://www.bmj.com/content/340/bmj.c181.long for guidelines on how to de-identify and prepare clinical data for publication. For a list of recommended repositories, please see https://journals.plos.org/plosone/s/recommended-repositories. You also have the option of uploading the data as Supporting Information files, but we would recommend depositing data directly to a data repository if possible.

Please update your Data Availability statement in the submission form accordingly.

4. If the reviewer comments include a recommendation to cite specific previously published works, please review and evaluate these publications to determine whether they are relevant and should be cited. There is no requirement to cite these works unless the editor has indicated otherwise.

Additional Editor Comments (if provided):

Reviewer #1:

[Note: HTML markup is below. Please do not edit.]

Reviewers' comments:

Reviewer's Responses to Questions

**Comments to the Author**

1. Does this manuscript meet PLOS Global Public Health’s publication criteria?

Reviewer #1: Yes

**********

2. Has the statistical analysis been performed appropriately and rigorously?

Reviewer #1: Yes

**********

3. Have the authors made all data underlying the findings in their manuscript fully available (please refer to the Data Availability Statement at the start of the manuscript PDF file)?

The PLOS Data policy

Reviewer #1: Yes

**********

4. Is the manuscript presented in an intelligible fashion and written in standard English?

Reviewer #1: Yes

**********

Reviewer #1: Introduction

1. Provides global/Tanzanian context with citations; builds rationale for study gap. Logical flow from CVD burden to lifestyle modification importance.

2. Could be concise; some repetition (e.g., CVD stats). Gap statement: Claims "paucity of research" in Tanzania, support with a brief literature search summary.

3. Grammar- "modest alcoholism" - "moderate alcohol consumption"; "significantly rise" - "significant rise".

4. Strengthen with 1-2 sentences on qualitative approaches' value in LMICs.

Materials and Methods

a) Sampling: n=10 is small but justified by saturation; however, describe recruitment variation (e.g., how ensured diversity in CVD types, age, gender? 60% female noted in results-move here?).

b) Data collection: Tool (interview guide) mentioned but not appended or described (e.g., sample questions). Interviews 20-30 min is a bit short for in-depth; justify. No mention of pilot testing or reflexivity (e.g., researcher bias as nurses/clinicians?).

c) Analysis: Steps outlined, but lacks detail on software (if used, e.g., NVivo), inter-rater reliability (e.g., how supervisors refined themes), or member checking for validity.

d) Study period: May-June 2024 in abstract; May-July in text take note of the inconsistency.

Results

Good; Links findings to literature; highlights implications (e.g., tailored education). Contrasts with global studies effectively.

a) Structure: Starts with summary but jumps between facilitators/barriers; maybe organize by theme?

b) Synthesis: Comparisons (e.g., to refs 24-28) are surface level; deepen (e.g., how Tanzanian cultural factors differ from Ethiopian study in ref 4?)

c) Novelty: Claims alignment but doesn't emphasize unique contributions (e.g., religious support in faith-based setting).

Conclusion

Good; Reiterates key findings and calls for action (e.g., personalized education),

a) Repetitive of abstract/discussion; too long for a concise wrap-up. Need to add implications for practice/policy.

Language and Style: A couple of errors (e.g., "life modification" - "lifestyle"; "i couldn’t" - "I couldn't"; run-on sentences). Proofread thoroughly or use editing tools. Enhance methodological details (rigor, reflexivity); fix inconsistencies (dates); deepen discussion synthesis.

**********

what does this mean?). If published, this will include your full peer review and any attached files.

**Do you want your identity to be public for this peer review?**  If you choose “no”, your identity will remain anonymous but your review may still be made public.

For information about this choice, including consent withdrawal, please see our Privacy Policy

Reviewer #1: **Yes:**  Doreen Mitaru

**********

[NOTE: If reviewer comments were submitted as an attachment file, they will be attached to this email and accessible via the submission site. Please log into your account, locate the manuscript record, and check for the action link "View Attachments". If this link does not appear, there are no attachment files.]

# Author comment: Author response to Decision Letter 1

**DOI:** 10.1371/journal.pgph.0007537.r002

20 Nov 2025

Attachment Submitted filename: Rebuttal letter.docx

# Editor report: Decision Letter 1

**DOI:** 10.1371/journal.pgph.0007537.r003

**Authors:** Hocheol Lee

3 Dec 2025

PGPH-D-25-02058R1

Facilitators and Barriers to Lifestyle Modification Practices among Cardiovascular Patients attending Cardiac Clinic at a Faith Based Hospital in Northern Tanzania

PLOS Global Public Health

Dear Dr. Mgala,

Thank you for submitting your manuscript to PLOS Global Public Health. After careful consideration, we feel that it has merit but does not fully meet PLOS Global Public Health’s publication criteria as it currently stands. Therefore, we invite you to submit a revised version of the manuscript that addresses the points raised during the review process.

Please submit your revised manuscript by Jan 17 2026 11:59PM. If you will need more time than this to complete your revisions, please reply to this message or contact the journal office at globalpubhealth@plos.org. When you're ready to submit your revision, log on to https://www.editorialmanager.com/pgph/ and select the 'Submissions Needing Revision' folder to locate your manuscript file.

Please include the following items when submitting your revised manuscript:

- A rebuttal letter that responds to each point raised by the editor and reviewer(s). You should upload this letter as a separate file labeled 'Response to Reviewers'.
- A marked-up copy of your manuscript that highlights changes made to the original version. You should upload this as a separate file labeled 'Revised Manuscript with Track Changes'.
- An unmarked version of your revised paper without tracked changes. You should upload this as a separate file labeled 'Manuscript'.

Guidelines for resubmitting your figure files are available below the reviewer comments at the end of this letter.

We look forward to receiving your revised manuscript.

Kind regards,

Hocheol Lee

Academic Editor

PLOS Global Public Health

Journal Requirements:

If the reviewer comments include a recommendation to cite specific previously published works, please review and evaluate these publications to determine whether they are relevant and should be cited. There is no requirement to cite these works unless the editor has indicated otherwise.

Additional Editor Comments (if provided):

Thank you for your submission. The reviewers recognize the relevance of your study in exploring lifestyle modification practices among cardiovascular patients in Tanzania, particularly in a faith-based healthcare setting. Your manuscript contributes to the growing body of qualitative research in LMICs and highlights culturally embedded barriers and facilitators to behavior change.

However, several important areas require substantial revision and elaboration before the manuscript can be considered for publication. Please address the following points in your revised manuscript:

[Note: HTML markup is below. Please do not edit.]

Reviewers' comments:

[NOTE: If reviewer comments were submitted as an attachment file, they will be attached to this email and accessible via the submission site. Please log into your account, locate the manuscript record, and check for the action link "View Attachments". If this link does not appear, there are no attachment files.]

# Author comment: Author response to Decision Letter 2

**DOI:** 10.1371/journal.pgph.0007537.r004

13 Jan 2026

Attachment Submitted filename: Response to Reviewers.doc

# Peer review: Decision Letter 2

**DOI:** 10.1371/journal.pgph.0007537.r005

**Authors:** Steve Zimmerman

1 Apr 2026

PGPH-D-25-02058R2

Facilitators and Barriers to Lifestyle Modification Practices among Cardiovascular Patients attending Cardiac Clinic at a Faith-Based Hospital in Northern Tanzania

PLOS Global Public Health

Dear Dr. Mgala,

Thank you for submitting your manuscript to PLOS Global Public Health. After careful consideration, we feel that it has merit but does not fully meet PLOS Global Public Health’s publication criteria as it currently stands. Therefore, we invite you to submit a revised version of the manuscript that addresses the points raised during the review process.

The manuscript has been evaluated by one reviewer, and their comments are attached.

Could you please revise the manuscript to carefully address the concerns raised?

Please submit your revised manuscript by May 01 2026 11:59PM. If you will need more time than this to complete your revisions, please reply to this message or contact the journal office at globalpubhealth@plos.org. When you're ready to submit your revision, log on to https://www.editorialmanager.com/pgph/ and select the 'Submissions Needing Revision' folder to locate your manuscript file.

Please include the following items when submitting your revised manuscript:

- A letter that responds to each point raised by the editor and reviewer(s). You should upload this letter as a separate file labeled 'Response to Reviewers'.
- A marked-up copy of your manuscript that highlights changes made to the original version. You should upload this as a separate file labeled 'Revised Manuscript with Track Changes'.
- An unmarked version of your revised paper without tracked changes. You should upload this as a separate file labeled 'Manuscript'.

Guidelines for resubmitting your figure files are available below the reviewer comments at the end of this letter.

As the corresponding author, your ORCID iD is verified in the submission system and will appear in the published article. PLOS supports the use of ORCID, and we encourage all coauthors to register for an ORCID iD and use it as well. Please encourage your coauthors to verify their ORCID iD within the submission system before final acceptance, as unverified ORCID iDs will not appear in the published article. *Only*  the individual author can complete the verification step; PLOS staff *cannot*  verify ORCID iDs on behalf of authors.

We look forward to receiving your revised manuscript.

Kind regards,

Steve Zimmerman, PhD

PLOS Staff Editor

**Journal Requirements:**

If the reviewer comments include a recommendation to cite specific previously published works, please review and evaluate these publications to determine whether they are relevant and should be cited. There is no requirement to cite these works unless the editor has indicated otherwise.

Please review your reference list to ensure that it is complete and correct. If you have cited papers that have been retracted, please include the rationale for doing so in the manuscript text, or remove these references and replace them with relevant current references. Any changes to the reference list should be mentioned in the rebuttal letter that accompanies your revised manuscript. If you need to cite a retracted article, indicate the article’s retracted status in the References list and also include a citation and full reference for the retraction notice.

**Additional Editor Comments (if provided):**

[Note: HTML markup is below. Please do not edit.]

Reviewers' comments:

Reviewer's Responses to Questions

**Comments to the Author**

Reviewer #1: (No Response)

**********

publication criteria?

Reviewer #1: Yes

**********

3. Has the statistical analysis been performed appropriately and rigorously?

Reviewer #1: Yes

**********

4. Have the authors made all data underlying the findings in their manuscript fully available (please refer to the Data Availability Statement at the start of the manuscript PDF file)?

The PLOS Data policy

Reviewer #1: Yes

**********

5. Is the manuscript presented in an intelligible fashion and written in standard English?

Reviewer #1: Yes

**********

**Reviewer #1:**  (No Response)

**********

what does this mean?). If published, this will include your full peer review and any attached files.

**Do you want your identity to be public for this peer review?**  If you choose “no”, your identity will remain anonymous but your review may still be made public.

For information about this choice, including consent withdrawal, please see our Privacy Policy

Reviewer #1: **Yes:**  Doreen Mitaru

**********

[NOTE: If reviewer comments were submitted as an attachment file, they will be attached to this email and accessible via the submission site. Please log into your account, locate the manuscript record, and check for the action link "View Attachments". If this link does not appear, there are no attachment files.]

**Figure Resubmissions:**

Attachment Submitted filename: Comments.docx

# Author comment: Author response to Decision Letter 3

**DOI:** 10.1371/journal.pgph.0007537.r006

14 May 2026

Attachment Submitted filename: Response_to_Reviewers_auresp_3.doc

# Peer review: Decision Letter 3

**DOI:** 10.1371/journal.pgph.0007537.r007

**Authors:** Ali Aahil Noorali

27 Jul 2026

PGPH-D-25-02058R3

Facilitators and Barriers to Lifestyle Modification Practices among Cardiovascular Patients attending Cardiac Clinic at a Referral Hospital in Northern Tanzania

PLOS Global Public Health

Dear Dr. Mgala,

Thank you for submitting your manuscript to PLOS Global Public Health. After careful consideration, we feel that it has merit but does not fully meet PLOS Global Public Health’s publication criteria as it currently stands. Therefore, we invite you to submit a revised version of the manuscript that addresses the points raised during the review process.

Please submit your revised manuscript by Sep 25 2026 11:59PM. If you will need more time than this to complete your revisions, please reply to this message or contact the journal office at globalpubhealth@plos.org. When you're ready to submit your revision, log on to https://www.editorialmanager.com/pgph/ and select the 'Submissions Needing Revision' folder to locate your manuscript file.

Please include the following items when submitting your revised manuscript:

- A letter that responds to each point raised by the editor and reviewer(s). You should upload this letter as a separate file labeled 'Response to Reviewers'.
- A marked-up copy of your manuscript that highlights changes made to the original version. You should upload this as a separate file labeled 'Revised Manuscript with Track Changes'.
- An unmarked version of your revised paper without tracked changes. You should upload this as a separate file labeled 'Manuscript'.

Guidelines for resubmitting your figure files are available below the reviewer comments at the end of this letter.

As the corresponding author, your ORCID iD is verified in the submission system and will appear in the published article. PLOS supports the use of ORCID, and we encourage all coauthors to register for an ORCID iD and use it as well. Please encourage your coauthors to verify their ORCID iD within the submission system before final acceptance, as unverified ORCID iDs will not appear in the published article. *Only*  the individual author can complete the verification step; PLOS staff *cannot*  verify ORCID iDs on behalf of authors.

We look forward to receiving your revised manuscript.

Kind regards,

Ali Aahil Noorali, MD, MPH, MBA

Guest Editor

PLOS Global Public Health

Journal Requirements:

If the reviewer comments include a recommendation to cite specific previously published works, please review and evaluate these publications to determine whether they are relevant and should be cited. There is no requirement to cite these works unless the editor has indicated otherwise.

Additional Editor Comments (if provided):

[Note: HTML markup is below. Please do not edit.]

Reviewers' comments:

Reviewer's Responses to Questions

**Comments to the Author**

Reviewer #2: (No Response)

Reviewer #3: All comments have been addressed

Reviewer #4: All comments have been addressed

Reviewer #5: All comments have been addressed

Reviewer #6: (No Response)

Reviewer #7: (No Response)

Reviewer #8: (No Response)

**********

publication criteria?

Reviewer #2: No

Reviewer #3: Yes

Reviewer #4: Yes

Reviewer #5: Yes

Reviewer #6: Yes

Reviewer #7: Yes

Reviewer #8: No

**********

3. Has the statistical analysis been performed appropriately and rigorously?

Reviewer #2: No

Reviewer #3: Yes

Reviewer #4: Yes

Reviewer #5: Yes

Reviewer #6: Yes

Reviewer #7: Yes

Reviewer #8: N/A

**********

4. Have the authors made all data underlying the findings in their manuscript fully available (please refer to the Data Availability Statement at the start of the manuscript PDF file)?

The PLOS Data policy

Reviewer #2: Yes

Reviewer #3: Yes

Reviewer #4: Yes

Reviewer #5: No

Reviewer #6: Yes

Reviewer #7: No

Reviewer #8: No

**********

5. Is the manuscript presented in an intelligible fashion and written in standard English?

Reviewer #2: No

Reviewer #3: Yes

Reviewer #4: Yes

Reviewer #5: Yes

Reviewer #6: Yes

Reviewer #7: Yes

Reviewer #8: No

**********

Reviewer #2: The title needs modification. The sample size is too small as the scope of the title is potentially broad. The methods used are not clear and appropriate.

Reviewer #3: This manuscript is written based on the qualitiative data. This is nicely written. The way of analyze the results is very much effective.

Reviewer #4: Thank you for addressing the previous reviewers' comments. The manuscript has improved considerably, particularly regarding the methodological description and the discussion. I have only a few minor comments that may further improve the clarity and consistency of the manuscript.

Introduction (lines 43–45): Please review the punctuation. Full stops appear to be missing after the references (2–4), LMICs (2,4), and 2030 (3,5).

Study area (line 99): Please ensure consistency between the response letter and the revised manuscript. The term "faith-based" still appears in the Study area section, although the response to the reviewers indicates that it was removed.

Study population and eligibility criteria (line 110): A full stop is missing at the end of the sentence.

Sample size and sampling procedure (lines 112–116): This paragraph appears to be redundant, as the same information is presented in greater detail immediately afterward. Please consider removing or integrating the duplicated content.

Sample size and sampling procedure (lines 128–135): The explanation regarding data saturation, participant recruitment, and purposive sampling is largely repeated. Please consider consolidating these paragraphs to improve conciseness.

Sample size and sampling procedure (line 127): A full stop is missing at the end of the sentence.

Data collection method and tool (lines 138–147): Some information overlaps with the subsequent Data collection procedure section. Please consider simplifying this paragraph by focusing on the interview guide and avoiding repetition.

Data collection procedure (lines 166–169): The justification for the interview duration contains repeated wording. Please consider removing the redundant text to improve readability.

Data analysis (line 177): Please consider rephrasing the sentence to avoid repetition of the word root (reflexivity / reflective), thereby improving readability.

Results (lines 228–232): Please review the punctuation separating the two main themes. A full stop appears to be missing after "...guidance from healthcare providers".

Results: Throughout the Results section, several quotations are presented consecutively without transition sentences or clear separation before the following quotation or sub-theme. Please consider adding brief transition sentences and reviewing the punctuation/formatting to improve the flow of the narrative.

Terminology: Please perform a final consistency check throughout the manuscript. Although the response letter indicates that previous terminology issues were addressed, expressions such as "CVDs patients" are still present.

Discussion (line 412): Please remove the duplicated expression "weight control" and revise the repeated use of the word "outcomes" within the same sentence.

Overall, the manuscript is well written and has substantially improved following the previous round of revisions. The remaining comments are primarily editorial and are intended to improve clarity, conciseness, and consistency.

Reviewer #5: The revised version seems considerably improved and is very close to being ready for acceptance. I don’t have any new methodological concerns, but minor copyediting is required before acceptance.

1. In the introduction on page 4, line 76, authors say that many studies “have primarily used qualitative approaches”. It should be quantitative approaches, as they are implying that these older studies measure and provide statistical clarity, while they are adding qualitative insight through this manuscript.

2. Page 6, lines 117 – 127 are repeated again in the paragraph below. Please remove one.

3. On page 14, line 308, the second theme, i.e., “Barriers to lifestyle modification practices” is labelled “Theme 4”. Instead, it should be Theme 2 as there are 2 final themes.

4. It seems like an editing note has been left in a participant quote “[what do you mean? Write it [e.g., correct information about lifestyle modification]”. Please correct this in line 361- 361. The same quote says “1 years” in line 361, which should be 1 year instead.

5. Som other issues that need correction:

• “CVDs-related deaths” in line 44, page 2, should be CVD-related deaths

• Please change “CVDs patients” on page 5, line 107 � CVD patients

• Page 14, line 306: “cooker” should be cook instead

• Line 351, page 16, has a typo i.e. “meals.t”

• Line 412, page 19, “weight control” is repeated twice

Reviewer #6: This manuscript addresses an important and underexplored topic in cardiovascular disease management in Tanzania. The qualitative findings are relevant and potentially valuable. However, the Discussion lacks sufficient critical analysis and interpretation, and the manuscript currently reads more as a descriptive report than a qualitative study offering deeper conceptual insights. I therefore recommend Major Revision before the manuscript can be considered for publication.

INTRODUCTION

Line 74-77: “Despite these efforts, the adherence to lifestyle modification among patients with CVDs remains suboptimal CVD, and many studies conducted in Tanzania and other African settings have primarily used qualitative approaches to measure the prevalence and associated factors.”

Did you mean to say quantitative here?

Line 81-83: This discrepancy between knowledge and practice suggests the presence of complex personal, social, cultural, and healthcare-related influences that cannot be fully explained through quantitative methods alone.

In public health and psychology, there is an extensive body of literature demonstrating that the knowledge-practice gap is a well-established phenomenon. Rather than attributing this discrepancy solely to "complex factors," the authors should acknowledge that knowledge is only one determinant of behavior, alongside factors such as self-efficacy, motivation, social norms, environmental constraints, access to resources, and health system influences. Please rephrase this sentence accordingly

\

Line 128-136 and 150-152: These statements are repeated earlier verbatim

RESULTS

Some themes can be grouped together, like religious and social support…

Results are reiterating participants’ quotes followed by author’s rewriting it, so consider adding one or two interpretive sentences after quotations to explain how the quotation illustrates the broader theme suggested by the authors.

DISCUSSION

This section provides little insight into why the findings occurred, how the Tanzanian context influences these findings…what this study contributes beyond previous literature, because right now it reads like a summary of results and if similar/dissimilar to previous studies, but no real in-depth analysis occurs. Needs substantial revision

Practical implications are too general/generic

LIMITATIONS

Many remain missing, like: Single referral hospital, potential selection bias, social desirability bias, possible recall bias, cross-sectional nature of interviews and limited transferability to other healthcare settings.

CONCLUSION

Reads like another summary and should include implications for clinical practice, future intervention development and future qualitative or mixed-methods research.

Reviewer #7: Hello authors. I comend you for your work. i hve some corrections that you should make before publishing

1. Results, participants description: the text states the duration since diagnosis ranged from six months to fifteen years, but the maximum in Table 1 is 14 years. Please correct.

2. Introduction, lines 45-47: "a high burden of non-communicable diseases (NCDs), with approximately 33%, whereby CVDs account for about 13% of its related deaths" is unclear. Specify 33% of what--presumably of all deaths?? Please rephrase

3. Under "Lack of guidance", the P3 quotation describes receiving explanations from doctors at the clinic, which contradicts the sub-theme. Either move it, or use the narrative to explain that experiences of guidance were mixed.

4 The quotation from P1 currently reads: "No, I have never received any (what do you mean? Write it (eg, correct information about lifestyle modification)". This appears to be an internal query between co-authors that was never resolved and has been carried into the clean manuscript as if it were participant speech.

5. The previous review asked for overlapping sub-themes to be merged, and the response letter states this was done. In the clean version, however, the content of the former "Personal health improvement" sub-theme has simply been moved under "Perceived benefits and outcomes", where the framing sentences and the P8 quotation now read as repetitive. In addition, the sub-theme "Motivation for lifestyle changes" is defined entirely by guidance from healthcare professionals and substantively duplicates "Medical advice and counselling". Either merge these two, or redefine "Motivation for lifestyle changes" so that it is analytically distinct.

6. The Discussion states "Many reported weight control, weight control, and other outcomes as outcomes of maintaining these healthy practices." Apart from the duplicated phrase, no participant quotation or results text mentions weight control; the reported outcomes were energy, breathlessness, blood sugar, and blood pressure. Please either evidence this claim in the Results or remove it, and correct the duplication.

Reviewer #8: PLOS Global Public Health

Manuscript Number: PGPH-D-25-02058R3

Facilitators and Barriers to Lifestyle Modification Practices among Cardiovascular Patients attending Cardiac Clinic at a Referral Hospital in Northern Tanzania

Benedicto Mgala, MSc. Cardiovascular Nursing Muhimbili University of Health and Allied Sciences Dar es Salaam, TANZANIA, UNITED REPUBLIC OF

Revision of the third version

The general topic of this study is very important for adding to research that is oriented toward improving behavioral intervention strategies in health systems. This review will focus mainly on the research approach especially in regard to the data collection instrument and the underlying behavioral theory that was added ex-post. I include general comments on the types of preventive behaviors, and a few grammar and placement comments.

Data collection instrument:

Thank you for including the interview guide as supplementary material which allows the reader to understand how the qualitative data was collected.

Based on the questions posed, the domains in the interview guide perhaps could be called more relevantly, “Influences on lifestyle modifications.” The questions do not seem to be oriented to identify lifestyle modification “practices” which are not specified in the methods (guide), results, or discussion sections. This point is also relevant to the title of the article.

The use of the term “lifestyle modifications,” as the main focus of the research question and interview guide, raises the issue as to how each interviewee understood and defined the term - “lifestyle modifications.” Based on information provided in the interview guide and manuscript, it is likely that neither the respondents nor the researchers knew which “lifestyle modification practices” were being discussed in the interviews, nor in the analysis. Their understanding could vary greatly between interviewees, and thus the answers would be in response to very different understandings of the question. If interviewees are not asked about specific behaviors, they are likely to give only very general responses to the questions. Some or most of the questions in the interview guide are leading questions (leading the respondent to provide a pre-ordained response), with the result that the final conclusions of the study closely track the language terminology utilized in the interview guide. Also, two of the eight interview questions are yes/no questions, with no evident follow-up inquiry to the answer.

Within the manuscript, the authors refer in passing to dietary adjustments, physical activity, smoking, and alcohol consumption in reference to “lifestyle modifications.” Previous research on these four behaviors in the literature has identified which specific lifestyle behaviors have been shown to be important (significantly associated) with improvements in CVD outcomes. These should be more fully discussed in the manuscript, with citations of this literature. This is important as the facilitators and barriers to a particular “lifestyle” behavior will vary based on which behavior is being considered. The available behavioral research on the four behaviors mentioned should be included in the introduction, as well as referred to in the discussion based on study results.

Underlying theory on behavior change:

The authors added a reference to Bandura’s Social Cognitive Theory when prompted by a reviewer. It is suggested to increase discussion on how that theory is relevant to this study design. In order to advance the field of behavioral analysis with this study, this reviewer suggests that current and future studies should be based on recognizable frameworks that have been developed on the basis of previous studies. Such frameworks can then be used as the basis for suggesting and then developing and testing more effective interventions to change behaviors.

For example, one of the earliest theories of health behavior, the “Health Belief Model,” is particularly relevant to analyzing and promoting health behaviors to prevent or treat illness. A more recent integrated model based on an in-depth review of literature on behavior change, is the COM-B model with six components: Capacities-internal (psychological [knowledge] and physical); Opportunity-external (psychological [ie. social norms, social support] and physical [i.e. environmental]); and Motivation-internal (reflexive [considering facts and proven science] and automatic [i.e. habit]), all leading to B – Behavior. See Michie et al. and their model called COM-B and the Behavior Change Wheel.* The key processes are first and foremost to identify what specific behavior needs to be changed. One then analyzes the influences on that behavior in terms of the COM-B model (with facilitators and barriers for each of the six components). Then for the intervention design, the model identifies the types of interventions that are best suited to modify or strengthen the major influences on the six components as analyzed; and finally identifies what public policy and teaching tools (i.e. laws, policies, manuals, guides, etc) can provide sustainability to each type of intervention identified.

* Susan Michie, Maartje M van Stralen & Robert West. The behavior change wheel: A new method for characterizing and designing behavior change interventions. Implementation Science 2011, 6:42

https://implementationscience.biomedcentral.com/articles/10.1186/1748-5908-6-42

http://www.behaviourchangewheel.com

Primary vs. secondary vs. tertiary prevention behaviors:

I suggest that this study provides the opportunity to differentiate types of preventive behaviors. Behaviors designed to ameliorate and slow progression of an existing illness, such as CVD, are tertiary prevention. On the other hand, primary prevention is practiced in presence of perfect health, so illness does not occur, such as healthy diet, exercise, consistent tooth-brushing etc. Secondary prevention is taking action to screen for and identify an illness that could have already begun, such as getting a dental check-up or blood-pressure screening or an annual physical exam.

Grammar and placement issues:

Line 53. Not clear “NCD’s account for 33%”. (of what?)

Line 87. Patients’. (add apostrophe)

Line 129. Clinic

Lines 129-131. Inclusion criteria should be moved to section on “Study population and eligibility criteria”

Final suggestion:

I hope the authors will continue to pursue this important line of research based on behavioral theory and a subsequently well-desiged and pretested research instrument. Health behavior change is kind of a new field of research and intervention, and it is important to build on what is already known to help the field develop properly.

**********

what does this mean?). If published, this will include your full peer review and any attached files.

**Do you want your identity to be public for this peer review?**  If you choose “no”, your identity will remain anonymous but your review may still be made public.

For information about this choice, including consent withdrawal, please see our Privacy Policy

Reviewer #2: No

Reviewer #3: **Yes:**  Md Abdullah Yusuf

Reviewer #4: No

Reviewer #5: No

Reviewer #6: No

Reviewer #7: No

Reviewer #8: **Yes:**  Laura Altobelli

**********

[NOTE: If reviewer comments were submitted as an attachment file, they will be attached to this email and accessible via the submission site. Please log into your account, locate the manuscript record, and check for the action link "View Attachments". If this link does not appear, there are no attachment files.]

Attachment Submitted filename: PeerReview.docx

# Author comment: Author response to Decision Letter 4

**DOI:** 10.1371/journal.pgph.0007537.r008

7 Aug 2026

Attachment Submitted filename: Response to Reviewers.docx

# Peer review: Decision Letter 4

**DOI:** 10.1371/journal.pgph.0007537.r009

**Authors:** Rabia Hussain

2 Oct 2026

PGPH-D-25-02058R4

Perceived Facilitators and Barriers Influencing Lifestyle Modification among Adults with Cardiovascular Disease Attending a Cardiac Clinic at a Referral Hospital in Northern Tanzania: An Exploratory Qualitative Study

PLOS Global Public Health

Dear Dr. Mgala,

Thank you for submitting your manuscript to PLOS Global Public Health. After careful consideration, we feel that it has merit but does not fully meet PLOS Global Public Health’s publication criteria as it currently stands. Therefore, we invite you to submit a revised version of the manuscript that addresses the points raised during the review process.

Please submit your revised manuscript by Nov 01 2026 11:59PM. If you will need more time than this to complete your revisions, please reply to this message or contact the journal office at globalpubhealth@plos.org. When you're ready to submit your revision, log on to https://www.editorialmanager.com/pgph/ and select the 'Submissions Needing Revision' folder to locate your manuscript file.

Please include the following items when submitting your revised manuscript:

- A letter that responds to each point raised by the editor and reviewer(s). You should upload this letter as a separate file labeled 'Response to Reviewers'.
- A marked-up copy of your manuscript that highlights changes made to the original version. You should upload this as a separate file labeled 'Revised Manuscript with Track Changes'.
- An unmarked version of your revised paper without tracked changes. You should upload this as a separate file labeled 'Manuscript'.

Guidelines for resubmitting your figure files are available below the reviewer comments at the end of this letter.

As the corresponding author, your ORCID iD is verified in the submission system and will appear in the published article. PLOS supports the use of ORCID, and we encourage all coauthors to register for an ORCID iD and use it as well. Please encourage your coauthors to verify their ORCID iD within the submission system before final acceptance, as unverified ORCID iDs will not appear in the published article. *Only*  the individual author can complete the verification step; PLOS staff *cannot*  verify ORCID iDs on behalf of authors.

We look forward to receiving your revised manuscript.

Kind regards,

Rabia Hussain, PhD

Academic Editor

PLOS Global Public Health

**Journal Requirements:**

If the reviewer comments include a recommendation to cite specific previously published works, please review and evaluate these publications to determine whether they are relevant and should be cited. There is no requirement to cite these works unless the editor has indicated otherwise.

Please review your reference list to ensure that it is complete and correct. If you have cited papers that have been retracted, please include the rationale for doing so in the manuscript text, or remove these references and replace them with relevant current references. Any changes to the reference list should be mentioned in the rebuttal letter that accompanies your revised manuscript. If you need to cite a retracted article, indicate the article’s retracted status in the References list and also include a citation and full reference for the retraction notice.

**Additional Editor Comments (if provided):**

There are a few comments that need to be addressed before the article can be accepted. Please address them all.

[Note: HTML markup is below. Please do not edit.]

Reviewers' comments:

Reviewer's Responses to Questions

**Comments to the Author**

Reviewer #3: All comments have been addressed

Reviewer #4: (No Response)

Reviewer #5: (No Response)

Reviewer #8: All comments have been addressed

**********

publication criteria?

Reviewer #3: Yes

Reviewer #4: Yes

Reviewer #5: Yes

Reviewer #8: Partly

**********

3. Has the statistical analysis been performed appropriately and rigorously?

Reviewer #3: Yes

Reviewer #4: Yes

Reviewer #5: N/A

Reviewer #8: N/A

**********

4. Have the authors made all data underlying the findings in their manuscript fully available (please refer to the Data Availability Statement at the start of the manuscript PDF file)?

The PLOS Data policy

Reviewer #3: Yes

Reviewer #4: Yes

Reviewer #5: No

Reviewer #8: No

**********

5. Is the manuscript presented in an intelligible fashion and written in standard English?

Reviewer #3: Yes

Reviewer #4: Yes

Reviewer #5: Yes

Reviewer #8: Yes

**********

**Reviewer #3:**  This manuscript is nicely revised in the sections of different sections.

The ethical issues are clearly stated according to the needful statement.

The methodology are described in details.

This manuscript can be published.

**Reviewer #4** : The punctuation issue identified in the previous round remains in the revised manuscript. Specifically:

Line 45: “strokes (2-4) Alarmingly” — a full stop is still missing after “(2-4)”.

Line 46: “countries (LMICs) (2,4) If effective” — a full stop is still missing after “(2,4)”.

Lines 46–47: “projected to increase to 23 million by 2030 (3,5) Tanzania is” — a full stop is still missing after “(3,5)”.

Line 119: “KCMC, a faith-based, zonal referral and teaching hospital...”

The term “faith-based” is still present in the revised manuscript, although the response letter explicitly states that it was removed. Please ensure that the correction indicated in the response letter is actually incorporated into the manuscript.

Line 140: “developed to explore participants’ perceived influences on lifestyle modification among.” The word “among” appears to be extraneous, leaving the sentence grammatically incomplete. Please revise this sentence for clarity and grammatical correctness.

Additionally, two corrections reported as completed in the response letter still appear to require attention in the revised manuscript:

“meals.t” should be corrected to “meals.”

“1 years with CVD” should be corrected to “1 year with CVD.”

The authors have satisfactorily addressed the main substantive and methodological concerns raised in the previous review. The manuscript has improved considerably. However, a few minor editorial inconsistencies remain, including corrections reported as completed in the response letter that are still present in the manuscript. Please address the specific points indicated above and carefully cross-check the final manuscript against the response letter. After these minor corrections, I believe the manuscript will be suitable for publication.

**Reviewer #5:**  The authors have addressed the substantive concerns raised in the previous round, and the manuscript is considerably improved. I do not have any further methodological concerns.

A few minor copyediting issues remain:

In the Results section, on page 16, line 352, the participant descriptor still reads “P1, 1 years with CVD.” Please change this to “P1, 1 year with CVD.”

The quotation under social and environmental barriers (page 15, line 340) still contains the typo “during meals.t.” Please correct this to “during meals.”

In the Abstract Objective, the phrase “among adults with cardiovascular patients attending the cardiac clinic” is incorrect. Please revise this to “among adults with cardiovascular disease attending the cardiac clinic” or similar wording.

**Reviewer #8:**  Line 140: the word "among". is dangling. please review English.

Line 140: guides or guide

Line 376: Gym membership should not be included as a valid 'financial constraint'. This is too easy an excuse: any exercise at all is useful and effective, including walking and floor exercises at home without equipment. If gym membership is to be included in the list of financial constraints, authors should comment that it is not a valid constraint to exercise.

Line 412: "partly aligns" : authors should be more adamant with the message that information and knowledge DO NOT themselves lead behavior change without other interventions.

**********

what does this mean?). If published, this will include your full peer review and any attached files.

**Do you want your identity to be public for this peer review?**  If you choose “no”, your identity will remain anonymous but your review may still be made public.

For information about this choice, including consent withdrawal, please see our Privacy Policy

Reviewer #3: **Yes:**  Prof. Dr. Md. Abdullah Yusuf

Reviewer #4: No

Reviewer #5: No

Reviewer #8: **Yes:**  Laura C Altobelli

**********

[NOTE: If reviewer comments were submitted as an attachment file, they will be attached to this email and accessible via the submission site. Please log into your account, locate the manuscript record, and check for the action link "View Attachments". If this link does not appear, there are no attachment files.]

**Figure Resubmissions:**

# Author comment: Author response to Decision Letter 5

**DOI:** 10.1371/journal.pgph.0007537.r010

3 Oct 2026

Attachment Submitted filename: Response_to_Reviewers_auresp_5.docx

# Editor report: Decision Letter 5

**DOI:** 10.1371/journal.pgph.0007537.r011

**Authors:** Rabia Hussain

11 Oct 2026

Perceived Facilitators and Barriers Influencing Lifestyle Modification among Adults with Cardiovascular Disease Attending a Cardiac Clinic at a Referral Hospital in Northern Tanzania: An Exploratory Qualitative Study

PGPH-D-25-02058R5

Dear Mgala,

We are pleased to inform you that your manuscript 'Perceived Facilitators and Barriers Influencing Lifestyle Modification among Adults with Cardiovascular Disease Attending a Cardiac Clinic at a Referral Hospital in Northern Tanzania: An Exploratory Qualitative Study ' has been provisionally accepted for publication in PLOS Global Public Health.

Before your manuscript can be formally accepted you will need to complete some formatting changes, which you will receive in a follow up email. A member of our team will be in touch with a set of requests.

Please note that your manuscript will not be scheduled for publication until you have made the required changes, so a swift response is appreciated.

IMPORTANT: The editorial review process is now complete. PLOS will only permit corrections to spelling, formatting or significant scientific errors from this point onwards. Requests for major changes, or any which affect the scientific understanding of your work, will cause delays to the publication date of your manuscript.

If your institution or institutions have a press office, please notify them about your upcoming paper to help maximize its impact. If they'll be preparing press materials, please inform our press team as soon as possible -- no later than 48 hours after receiving the formal acceptance. Your manuscript will remain under strict press embargo until 2 pm Eastern Time on the date of publication. For more information, please contact globalpubhealth@plos.org.

Thank you again for supporting Open Access publishing; we are looking forward to publishing your work in PLOS Global Public Health.

Best regards,

Rabia Hussain, PhD

Academic Editor

PLOS Global Public Health

***********************************************************

Reviewer Comments (if any, and for reference):
